# Early Alterations in Operant Performance and Prominent Huntingtin Aggregation in a Congenic F344 Rat Line of the Classical CAG_n51trunc_ Model of Huntington Disease

**DOI:** 10.3389/fnins.2018.00011

**Published:** 2018-01-25

**Authors:** Anne-Christine Plank, Fabio Canneva, Kerstin A. Raber, Yvonne K. Urbach, Julia Dobner, Maja Puchades, Jan G. Bjaalie, Clarissa Gillmann, Tobias Bäuerle, Olaf Riess, Hoa H. P. Nguyen, Stephan von Hörsten

**Affiliations:** ^1^Experimental Therapy, Preclinical Experimental Center, University Clinics Erlangen, Erlangen, Germany; ^2^Neural Systems Laboratory, Institute of Basic Medical Sciences, University of Oslo, Oslo, Norway; ^3^Preclinical Imaging Platform Erlangen, Institute of Radiology, University Clinics Erlangen, Erlangen, Germany; ^4^Institute of Medical Genetics and Applied Genomics, University Clinics Tuebingen, Tuebingen, Germany

**Keywords:** Huntington disease, transgenic rat model, F344 rat, behavioral phenotyping, operant conditioning, huntingtin aggregates

## Abstract

The transgenic rat model of Huntington disease expressing a fragment of mutant HTT (tgHD rat) has been thoroughly characterized and reproduces hallmark symptoms of human adult-onset HD. Pursuing the optimization of this model for evaluation of translational therapeutic approaches, the F344 inbred rat strain was considered as advantageous genetic background for the expression of the HD transgenic construct. In the present study, a novel congenic line of the SPRDtgHD transgenic model of HD, carrying 51 CAG repeats, was generated on the F344 rat genetic background. To assess the behavioral phenotype, classical assays investigating motor function, emotion, and sensorimotor gating were applied, along with automated screening of metabolic and activity parameters as well as operant conditioning tasks. The neuropathological phenotype was analyzed by immunohistochemistry and *ex vivo* magnetic resonance imaging. F344tgHD rats displayed markedly reduced anxiety-like behavior in the social interaction test and elevated impulsivity traits already at 3 months of age. Neuropathologically, reduced striatal volume and pronounced aggregation of mutant huntingtin in several brain regions were detected at later disease stage. In conclusion, the congenic F344tgHD model reproduces key aspects of the human HD phenotype, substantiating its value for translational therapeutic approaches.

## Introduction

Huntington disease (HD) is an autosomal dominantly inherited, neurodegenerative disorder caused by the expansion of a trinucleotide repeat (>36 CAG) in exon 1 of the huntingtin gene (*HTT*) on chromosome 4 (The Huntington's Disease Collaborative Research Group, 1993). The resulting aberrant polyglutamine (polyQ) strand elongation in the huntingtin protein induces progressive neuronal atrophy and cell loss in several brain regions, including cerebral cortex, thalamus, hypothalamus and basal ganglia with pronounced degeneration of striatal medium spiny neurons (MSN) (Vonsattel et al., 1985, 2008). Brains of HD patients accordingly display significant volume reductions (Rosas et al., 2003), enlargement of the lateral ventricles (LV) as well as neuronal intranuclear inclusions and cytoplasmic aggregates enriched in mutated huntingtin protein (mHTT) (DiFiglia et al., 1997). Clinically, HD hallmark symptoms comprise motor dysfunction, psychiatric disturbance and cognitive impairment. Movement abnormalities such as chorea and incoordination might be preceded by subtle personality changes and deficits in mental processes, comprising executive functions, attention and memory (Bates et al., 2002). In general, impairment of executive functions is frequently associated with reduced self-control manifesting in impulsive and compulsive behavior (Bates et al., 2002; Beglinger et al., 2008). At later disease stages, cognitive dysfunctions become evident in specific tests requiring planning, flexibility, concentration and inhibition of inappropriate responses (Montoya et al., 2006). A progressive aggravation of memory deficits ends up in subcortical dementia syndrome, characterized by slower processing of information, decreased motivation, depression and personality changes (Paulsen et al., 1995). To date, HD is not curable and leads to death after onset of symptoms in midlife within a period of 15–20 years.

Consequently, animal models providing high face and predictive validity are essential for further investigation of HD pathomechanisms and solid evaluation of potential therapies. To date, a wide range of HD models generated in various species is available, with rodents being the most commonly used. Diverse genetically engineered mouse models provide a valuable tool for neuropathological, behavioral and experimental therapeutic studies (for review see Pouladi et al., 2013). However, modeling neurodegenerative disorders in *Rattus norvegicus* offers several advantages, such as higher similarity of rat and human brain organization, convenient imaging and surgery due to larger size, and more complex behavioral phenotypes (Urbach et al., 2010; Carreira et al., 2015). For instance, more sophisticated behavioral tests of learning and memory can be applied in this species compared to mouse.

Our group pioneered the publication of the first Huntington disease rat model in 2003 (von Hörsten et al., 2003). The tgHD (CAG_n51trunc_) rat was generated by microinjection of a 1962 base pair rat HD cDNA fragment carrying 51 CAG repeats of the human *HTT* gene under the control of the rat *Htt* promoter. Transgene expression produces a mutant amino-terminal fragment corresponding to 22% of the full-length huntingtin protein (von Hörsten et al., 2003). The transgenic line was established in the genetic background of the Sprague-Dawley (SPRD) outbred rat strain and has been thoroughly characterized over the past years, demonstrating a slowly progressing phenotype that mirrors key symptoms of adult-onset HD. Motor dysfunctions reported in this model span hyperactivity (starting at 2 months), deficits in coordination and balance (starting at 6 months), gait disturbances (starting at 12 months) as well as opisthotonus-like movements (17 months) and chorea (20 months) (von Hörsten et al., 2003; Cao et al., 2006; Nguyen et al., 2006; Bode et al., 2009; Ortiz et al., 2012; Zeef et al., 2012b, 2014). Emotional alterations were found to precede motor symptoms in tgHD rats, measured as reduced anxiety-like behavior in the elevated plus maze and social interaction test of anxiety from 2 months of age (von Hörsten et al., 2003; Nguyen et al., 2006; Kirch et al., 2013). Moreover, tgHD rats displayed increased impulsivity traits when performing specific operant tasks (Cao et al., 2006; Temel et al., 2006; Urbach et al., 2014; El Massioui et al., 2016). First signs of progressive cognitive decline were detected between 6 and 9 months of age in different maze tests (Nguyen et al., 2006; Kirch et al., 2013) and at older ages in object recognition (Zeef et al., 2012a) and choice reaction time tasks (Cao et al., 2006; Kantor et al., 2006; Temel et al., 2006). Further key features of the adult-onset, progressive HD phenotype were reproduced in this model with regard to neuropathological symptoms. N-terminal (m)HTT containing neuropil aggregates and nuclear inclusions were found in the basal ganglia of animals aged 1 year and older (von Hörsten et al., 2003; Petrasch-Parwez et al., 2007; Kirch et al., 2013), and polyQ-containing aggregates as well as recruitment sites were detected in basal ganglia, deep cortical layers, thalamic nuclei, hypothalamus and substantia nigra *pars compacta* from the age of 9 months (Nguyen et al., 2006). Striatal atrophy and associated enlargement of LV became evident from 12 months of age (Kantor et al., 2006; Nguyen et al., 2006). Finally, several neurophysiological parameters such as levels of distinct hormones, neurotransmitters and receptors were found to be altered in transgenic animals (von Hörsten et al., 2003; Bauer et al., 2005; Bode et al., 2008, 2009; Cong et al., 2012; Jahanshahi et al., 2013), who also displayed decreased body weight gain and reduced life expectancy compared to wild-type controls (von Hörsten et al., 2003).

Taken together, the phenotype discovered in the tgHD (CAG_n51trunc_) rats convincingly mirrors human pathology of adult-onset HD, substantiating their value for preclinical therapeutic studies. For this reason, we sought to optimize this model by implementing the same transgene expression in the F344 inbred rat strain (F344tgHD). Using inbred strains for disease modeling offers several benefits regarding reliability and reproducibility of the results. Besides, a practical advantage is given by the fact that F344 rats are of smaller, leaner size, and therefore more suitable for *in vivo* MR-screening. In the present study, we describe the comprehensive phenotyping of this new congenic line, comprising classical behavioral tests as well as automated metabolic measurements and intra-home cage-like cognitive testing. Neuropathological features of HD are also addressed via classical immunohistochemistry and *ex vivo* magnetic resonance imaging (MRI). An early behavioral phenotype was detected in F344tgHD rats, with markedly reduced anxiety-like behavior in the social interaction test and elevated impulsivity traits in operant tasks observed in 3-month-old animals. Adult F344tgHD rats displayed pronounced aggregation of mutated huntingtin in several brain regions and reduced striatal volume. In conclusion, our results substantiate the findings published on the model in the SPRD background, confirming its face validity based on the underlying genetic construct.

## Materials and methods

### Animals

#### Generation of the congenic F344tgHD line

The F344tgHD congenic line was derived from a colony of Sprague-Dawley transgenic HD rats expressing 727 amino acids of the HD gene with 51 CAG repeats (cDNA position 324–2321, corresponding to 22% of full length) under the control of the rat *Htt* promoter (von Hörsten et al., 2003). Generation of the congenic strain was initiated following the speed congenic approach (Wakeland et al., 1997) by crossing SPRDtgHD homozygous females with a F344 wild-type male rat to fix the Y chromosome of the F344 background. Male F1 rats were then backcrossed to F344 females. Heterozygosity of the HD transgene of the resulting N2 males was confirmed via probe-based real-time PCR on DNA extracted from tail tip biopsies. The genetic background of N2 heterozygous males was determined using 100 polymorphic microsatellite markers with an intermarker distance of approximately 20 cM covering all chromosomes, as previously described (Frerker et al., 2009). The N2 male with the highest proportion of F344 background was selected for the next cross, and this scheme was applied for each generation until N5. Finally, an N5 male and an N5 female, both homozygous for the F344 background, were intermated to produce F344tgHD founders. The tgHD congenic F344 strain is maintained via brother × sister mating at the preclinical research center of the University Clinics Erlangen. Regular back-crossing on wild-type F344 rats is routinely performed every second year in order to avoid genetic-drift within the inbred F344 donor genome.

#### Genotyping

Genotyping was performed after weaning via probe-based real-time PCR (TaqMan PCR) on genomic DNA extracted from tail biopsy tissue (Qiagen, Germany) (primer: FW: 5′-AGCCCCATTCATTACCTTGCT-3′; RV: 5′-ATCAAGCTTATCGATACCGTC-3′; probe: 5′FAM-CTAAGTGGCGCTGCGTAGTGCGAA-3′Bhq). Ct values were normalized referring to ß-actin (FW: 5′-AGCCATGTACGTAGCCATCCA-3′; RV: 5′-TCTCCGGAGTCCATCACAATG-3′; probe: 5′VIC-TGTCCCTGTATGCCTCTGGTCGTACCAC-3′Bhq). Internal controls for each genotype served as reference for relative copy number estimation per rat.

#### Experimental subjects

In the present study, two cohorts of age-matched male homozygous transgenic (tg) and wild-type (wt) littermates were dedicated to behavioral phenotyping. Cohort 1 consisted of 9 wt and 11 tg rats while cohort 2 comprised 6 wt and 6 tg rats. Brains collected from cohort 2 at the age of 15 months were dedicated to *ex vivo* MR imaging studies. For immunohistochemistry, brains of 21-month-old female homozygous transgenic and wild-type littermates were analyzed.

Rats were housed in groups of four per cage with food (Ssniff lab chow pellets, Germany) and tap water available *ad libitum*, under controlled temperature- (22 ± 1°C), humidity- (55 ± 10% rel. humidity) and lighting- (12 h light/dark cycle) conditions.

All research and animal care procedures were performed in compliance with international animal welfare standards and approved by the district governments of Lower Saxony, Hannover, Germany (Laves #33-42502-05/931) and Lower Franconia, Würzburg, Bavaria, Germany (RegUFr #55.2-2532-2-223).

### Behavioral assessment

#### Experimental design

Experimental cohort 1 was repeatedly analyzed in the PhenoMaster System and in classical behavioral tests, which were selected on the basis of our previous work with SPRDtgHD rats (von Hörsten et al., 2003; Urbach et al., 2014). Cohort 2 underwent cognitive assessment in the automated operant conditioning system at the age of 3, 6, and 12 months. Cognitive, accelerod and social interaction tests were performed during the dark phase, while PPI and open field tests were performed during the light phase of the light/dark cycle. The PhenoMaster experiments lasted 72 h, i.e., three full light/dark cycles. It should be noted here that it will be important to investigate potential sex differences, as reported for the SPRDtgHD model (Bode et al., 2008), in future studies on female F344tgHD rats.

#### General health

The general health status of each animal was monitored regularly following previously published protocols (Karl et al., 2003; Urbach et al., 2010): parameters such as morphology, body weight, neurological reflexes and sensory function (hearing, vision) were included in the screening protocol.

#### Accelerod test

Motor coordination and balance of experimental animals was investigated using an Accelerod for rats (TSE Systems GmbH, Germany). The four-place apparatus consisted of a base platform and a rotating rod of 6 cm diameter with a non-skid surface and modifiable speed, monitored by a bar-graph type of speed indicator placed on the front panel. Rats were trained in four trials per day on 3 consecutive days at a constant speed of 12 rpm, in order to attain a steady baseline level of performance. Unmotivated animals not reaching such level were excluded from the following accelerod test. This test has been shown to be more sensitive than the rotarod to detect motor function deficits (Bogo et al., 1981) and to produce more consistent results (Jones and Roberts, 1968). On the day of accelerod testing, rats were placed on the rod at an initial speed of 4 rpm, which was then accelerated up to 40 rpm over 5 min. The performance of the rats was measured as max. rpm on the rod reached before falling and the test was repeated after 1 h. Animals were investigated the age of 3, 6, 9, and 15 months. Data were analyzed as average performance in both tests per animal and age point, and subsequently subjected to two-way repeated measures Anova.

#### Open field test

Spontaneous locomotion as well as anxiety-like behavior in a novel environment were evaluated in an open field task. A black square box subdivided into four separate arenas (50 cm × 50 cm each) was used to test four rats simultaneously; transmission of olfactory cues was prevented by thorough cleaning with ethanol (70%) after each session. Rats aged 6 months were placed in the center of the arena and allowed to explore it freely for 5 min while locomotor activity was recorded via a camera and dedicated tracking software (Viewer®, Biobserve). For analysis, each arena was virtually split into the following areas: “corners,” “wall zones,” and “center.” Distinct behavioral parameters attributable to spontaneous locomotion (e.g., distance traveled) or to anxiety-like behavior (e.g., time spent in center) were assessed for each animal. Two animals had to be excluded due lack of exploratory behavior (complete immobility for ≥60% of test duration), resulting in a remaining n of 4 wt vs. 6 tg animals. Data were analyzed applying unpaired *t*-tests (parametric, two-tailed).

#### Social interaction (SI) test of anxiety

The SI test was performed as described previously (Urbach et al., 2014) at the age of 2, 4, 6, and 8 months. Two genotype-matched rats (wt: *n* = 8, tg: *n* = 10) from different social groups were placed in a squared open field arena (50 cm × 50 cm) enclosed by a soundproof box and lit by a red photo bulb providing 1.3 lux. Behavior was monitored for 10 min by a video camera located inside the sound isolation box. Testing started 1 h after onset of the dark phase, and the following parameters were scored: (1) social interaction, defined as the time spent by the pair of rats sniffing, following, crawling under and over each other (passive body contact such as resting and sleeping was not recorded); (2) self-grooming, as the time spent by each rat grooming itself. Cumulative data per testing pair and parameter were statistically analyzed via two-way Anova and uncorrected Fisher's LSD test for *post-hoc* comparisons.

#### Acoustic startle response (ASR) and prepulse inhibition (PPI) of the startle

A four-place startle response system (TSE Systems GmbH, Germany) was used as previously described (Urbach et al., 2014). Each rat was placed in a wire-mesh cage (27 cm × 9 cm × 10 cm) that was subsequently fixed on a piezo-accelerometer in a sound-attenuated chamber, equipped with two loudspeakers located at both sides of the animal. Rats were investigated monthly at the age of 1–9 months. Test sessions were composed of several types of trials presented over a constant white background noise of 68 dB. A habituation phase of 2 min was followed by 10 repetitions of a standard startling noise (120 dB, 20 ms) presented with an inter-trial interval (ITI) of 6–12 s, in order to establish the baseline startle reaction of the experimental animals. For the assessment of PPI rats were subsequently exposed to three different types of trial, presented in a random order: (1) PPI trials, in which a startling noise (120 dB, 20 ms) was preceded by a prepulse of 72, 76, 80, or 84 dB (20, 100 ms latency to startle noise, 10 repetitions per prepulse intensity); (2) prepulses only (10 repetitions per prepulse intensity); (3) startle noise only (15 repetitions). All trials were separated by a 6–12 s ITI and a constant 68 dB white background noise was presented during the experiment. Animals received a total number of 77 trials within 14 min of testing. The median of ASR magnitudes obtained during the first 10 startling stimuli was calculated per animal and expressed as arbitrary units. PPI was then calculated as the percent inhibition of the ASR in the presence of a prepulse:

$$\begin{array}{l} \%\text{PPI}=100-\left[{\left(\text{MaxG}/\text{median ASR}\right)}^{*}100\right] \end{array}$$

Intra-individual PPI values obtained for the different prepulse intensities were analyzed via two-way repeated measures Anova, revealing that the prepulse of 84 dB inhibited the ASR most efficiently. Data collected for this measure at the age of 3, 6, and 9 months were then further analyzed with a two-way repeated measures Anova, with “genotype” and “age” as the between and within group factors, to investigate the age-dependent variation of the PPI responsiveness in the two genotypes.

#### Automated behavioral and metabolic phenotyping

##### Apparatus and experimental setup

For the assessment of circadian patterns of locomotor activity, ingestion behavior and indirect calorimetric parameters per rat in a home cage environment, a 12-place PhenoMaster System (TSE Systems GmbH, Germany) was used as described in detail before (Urbach et al., 2014). Briefly, spontaneous locomotion (XY plane) and rearing events (Z plane) were detected by infrared light beam frames surrounding each experimental cage (based on standard type IV cages, filled with defined amount of sawdust). Two dedicated weighing sensors integrated into the cage lid registered *ad libitum* water (tap water) and food (standard lab chow) consumption of the animal. Respiratory gas analysis (push respirometry) via an O_2_/CO_2_ gas sensor pair allowed the automated calculation of O_2_ consumption, CO_2_ production, respiratory exchange rate (RER = VCO_2_ [ml/h/kg BW]/VO_2_ [ml/h/kg BW]) and energy expenditure (H = [kcal/h/kg BW]) for each animal (performed by TSE PhenoMaster Software, version 4.4.6). The gas sensor pair was calibrated with calibration gas mixtures (CO_2_, 0.05%, O_2_ 20.90% ± 0.1%, in N_2_; CO_2_ 0.95% ± 1%, O_2_ 20.00% ± 0.1%, in N_2_) before each test session. Experiments were performed in a dedicated room under standard housing and light cycle conditions (described above). Rats were individually monitored in test sessions lasting 72 h, which were repeated in monthly intervals at the age of 1–9 months. After each experiment, all animals were re-unified in their original social groups.

#### Data processing and analysis

All data recorded during one test session were extracted at 20 min intervals. Intra-individual longitudinal analysis across all monitored age-points was achieved by compacting collected data as follows: for each 72 h—acquisition interval, average (RER, VCO_2_, VO_2_, and heat production) or cumulative (XT+YT, Z, food-/water consumption) values were calculated by separating data derived from the dark and light phases of the daily cycle. As habituation processes might bias data derived from the first (incomplete) light and dark phase/12 h of measurement, only data collected during the second and third respective day phase were pooled. Results were analyzed (split by day phase) using two-way repeated measures Anova, with the factor genotype representing the inter-individual factor and parameter across time points the intra-individual factor, and uncorrected Fisher's LSD test for *post-hoc* comparisons.

#### Operant learning

##### Apparatus and experimental setup

Twelve computer-controlled operant walls (TSE Systems GmbH, Germany) were used to investigate 6 wild-type and 6 transgenic rats in different operant conditioning tasks. One unit consists of two retractable levers positioned on either side of a central food crib, covered with a clear Perspex panel hinged at the top, so that the animal could push it open to retrieve the food reward (dustless precision pellets, 45 mg, Bio-Serv, Frenchtown, NJ, USA). Light stimuli can be given via five different light sources, including a house light at the top left corner, 3 signaling lights, positioned above each lever (red and green, respectively) and the food crib (white), and a yellow light inside the food compartment for indicating the presence of a food pellet. A dedicated software (*IntelliMaze* v. 4.0.0, NewBehavior) allowed to design different operant schedules, which were run automatically and independently on each wall during the dark phase of the light cycle.

Experiments were performed in a dedicated room under standard housing and light cycle conditions at 5–6 days per week. Each operant wall was located in an experimental cage (as described for the PhenoMaster system) providing a home cage-like environment, with tap water available *ad libitum*. Animals were placed into their dedicated testing cages at the end of the light phase; the first test session was started 2 h past onset of the dark phase. After each testing night, rats were re-unified in their original social groups. Animals were food-restricted during the testing phases and kept on ≥85% of their *ad libitum* body weight by daily temporary feeding with standard lab chow in their home cages.

Initially, rats were habituated to the operant wall setup by an adaptation schedule, delivering one food pellet as immediate reward for using one lever, or one free food pellet every 10 min if no lever was used. Ten pellets had to be earned by lever pressing before an animal was switched to the next schedule, offering a maximum of 100 pellets exclusively after lever pressing. This session was repeated 2 more times during the testing night (inter-session interval: 3 h). Only rats that managed to reliably collect at least 100 pellets within a 30-min session were subsequently tested in different operant conditioning tasks.

##### Randomized alternation

Associative conditioning can be trained by signaling to the animals which of the available response devices should be operated. For this purpose, a randomized alternation task that also inquires attention and motivation was conducted in this study at the age of 3, 6, and 12 months. Both levers were presented during test sessions, and one of the associated stimulus lights was illuminated per trial in a random sequence, indicating which lever had to be pressed to obtain a reward. A correct choice was followed by the delivery of one food pellet, and the next trial was started after retrieval of the pellet and an inter-trial interval (ITI) of 10 s. Wrong side lever presses as well as lacking response for 90 s induced a switch to the ITI and subsequent trial without reward delivery. A total number of 100 trials was given per session, with three sessions per night (inter-session interval: 3 h). Animals were tested at the age of 3, 6, and 12 months. The following parameters were determined: successful trials, lever presses during the ITI, time to complete session and side bias index which was calculated as follows:

$$\begin{array}{l} \left(\text{lever press on right lever}/\text{total lever presses}\right)-0.5=X\\ \%\text{ side bias}={\left(\text{absolute value of X}/0.5\right)}^{*}100 \end{array}$$

Data analyzed as mean performance per test session or testing night were subjected to two-way repeated measures Anova and uncorrected Fisher's LSD test for *post-hoc* comparisons.

##### Differential reinforcement of low rates of responding (DRL) task

The DRL task is based on Skinner's demonstration that the time between responses is a conditionable dimension of behavior (Pizzo et al., 2009). It is an established test for impulsivity traits, assessing the animals' capacity to withhold responding for a defined time interval in order to obtain a reward. The DRL criteria applied in this study were chosen based on the work of Pizzo et al. (2009).

Only the left lever was presented during test sessions, and the corresponding stimulus light was illuminated at the beginning of each trial. It was switched off with the first lever press and a timer, set to a defined inter-response time interval (IRT), was started. Upon expiration of the IRT, the stimulus light was switched on again until the second lever press was performed, which resulted in delivery of a single food pellet and completion of this trial. Any premature response given within the IRT reset the timer to zero and was recorded as an error. Rats were tested in two sessions per night, each lasting until either a preset timeout or the maximum number of successful trials (limited to 115) was reached. An IRT of 15 s was applied for two testing nights and subsequently increased to 30 s (3 nights) and 72 s (9 nights, with one session only due to lacking motivation in second sessions).

The number of successful trials and premature responses as well as the inter-response time were recorded per test session and animal. Performance efficiency was defined as ratio of reinforced to total responses (El Massioui et al., 2016). The relative number of burst responses (latency to second lever press <1 s) was calculated as the ratio of burst responses to total premature responses. The experimental cohort was tested in the DRL task at 3 and 6 months of age. The mean performance per test session was calculated and data were analyzed via two-way repeated measures Anova, followed by uncorrected Fisher's LSD test for *post-hoc* comparisons.

##### Progressive ratio

In contrast to the suppressive effect of the DRL schedule on behavior, the Progressive Ratio (PR) test induces high rates of responding. It is designed to measure the animals' motivation by forcing them to put progressively increasing physical effort into receiving a single food pellet as reward. The level of motivation is determined as the highest workload performed per test session.

Again, only one of the two levers was presented and had to be operated consecutively (time lag between two lever presses <2 s) to obtain a single reward. The number of required lever presses per food pellet was increased according to a fixed ratio (FR) schedule, with three repetitions of each FR performed successively as prerequisite for a switch to the following FR (i.e., 2, 2, 2, – 4, 4, 4, – 8, 8, 8 etc.). Eventually, the animals reached a point at which they stopped working – the “breakpoint,” defined as the highest FR value at which a successful triplicate response was recorded. Besides, the following parameters were measured: mean perseverative lever presses (the lever presses recorded after delivery and before collection of the food pellet, but which did not count toward the ratio requirement), calculated as total nr of perseverative lever presses / total nr of rewards earned, and median lever press duration per animal. All time measurements were recorded to the nearest 0.01 s. Results obtained at the age of 3, 6, and 12 months were analyzed via two-way repeated measures Anova and uncorrected Fisher's LSD test for *post-hoc* comparisons.

### Statistical analysis

Statistical analyses were performed using GraphPad Prism software (version 7). The D'Agostino and Pearson omnibus normality test (cohort 1) or Shapiro-Wilk normality test (cohort 2) confirmed that data were not inconsistent with a Gaussian distribution. Based on these results, parametric tests were applied for each data set, mainly two-factorial ANOVA with “genotype” defined as the between-subject factor and repeated measurements on one or more factors, depending on the behavioral test design. A critical value for significance of *p* < 0.05 was used throughout the study. The Uncorrected Fisher's LSD test was used for *post-hoc* comparison. All data represent means ± SEM.

### Tissue preparation and analyses

Rats were deeply anesthetized with pentobarbital (40 mg/kg BW, i.p.) and transcardially perfused with ice-cold saline solution (0.2 M Phosphate buffer, supplemented with 137 mM NaCl, 3 mM KCl and 6 mM NaHCO_3_). For immunohistochemistry, the brains were isolated, post-fixed overnight in 4% paraformaldehyde solution (PFA; pH 7.5), transferred to 30% sucrose in phosphate buffered saline (PBS; Biochrom AG, Germany) for 48 h and finally snap-frozen in isopentane for 30 s at −60°C and stored at −80°C until use. For *ex vivo* imaging studies, brains were removed and fixed in 4% formalin solution.

#### Immunohistochemistry

Frozen, fixed brains were sliced into 40 μm-thick coronal sections on a cryostat (CM3050S, Leica Biosystems, Germany). The sections were stained using a free-floating immunohistochemistry (IHC) procedure. PBS or PBS-T (PBS containing 0.2% TritonX-100) was used throughout all passages and incubations were performed at room temperature, unless otherwise specified. Briefly, tissue was washed with PBS and permeabilized with PBS-T. For staining with the antibody 2B7, sections were treated with 0.25% glutaraldehyde for 10 min, rinsed and subjected to antigen retrieval by application of >95% formic acid for 10 min. Endogenous peroxidases were blocked by treatment with 0.3% hydrogen peroxide for 20 min. Subsequently, sections were rinsed and treated with 5% normal donkey serum (nds, Jackson ImmunoResearch, Europe) in PBS-T for 1 h. Incubation with primary antibodies (monoclonal mouse-anti-hHTT aa1, KY-2B7-A2, Novartis, Germany; sheep-anti-huntingtin S829, kind gift of Gillian Bates, King's College, London, UK) diluted in REAL antibody diluent® (Dako, Agilent, USA) was performed overnight at 4°C. The following day sections were washed and incubated for 1 h with secondary antibody (biotinylated donkey-anti-mouse IgG, Santa Cruz, USA) diluted in 5% nds. Finally, tissue was rinsed and incubated for 30 min with VECSTASTAIN® ABC reagent (Vector Laboratories, UK). Immunoreactivity was visualized applying Ni-enhanced DAB (3,3′-diaminobenzidine) reagent (Vector Laboratories, UK) for single stainings, or DAB only for double staining with anti-NeuN antibody. Sections dedicated to double staining were subsequently rinsed, treated with avidin and biotin blocking reagent (10 min each) and incubated in 5% nds for 1 h and antibody solution (rabbit-anti-NeuN (EPR12763) monoclonal antibody, Abcam, UK) at 4°C overnight. For visualization of NeuN immunoreactivity, HRP-Green solution (42 life sciences, Germany) instead of DAB was used.

Finally, sections were mounted on slides, air-dried overnight, dehydrated and coverslipped with Fluka DPX mountant for histology (Sigma-Aldrich, USA). Pictures were taken using a Zeiss Axioscan Z1 slide scanner running Zeiss Zen Software (Carl Zeiss MicroImaging, Germany) with a 20x objective yielding images with a resolution of 0.22 μm/pixel.

#### *Ex vivo* magnetic resonance imaging

Formalin-fixed brains were embedded in 2% agarose and scanned on a preclinical ultra-high field magnetic resonance imaging system (7 Tesla ClinScan 70/30, Bruker, Germany). Morphologic images were obtained using a T1-weighted turboflash magnetization-prepared rapid gradient echo sequence [repetition time (TR)/echo time (TE): 50/2.5 ms, averages (av): 8, acquisition time (TA): 3:30 h]. Voxel-specific T1 relaxation times were mapped using a fast low angle shot (FLASH) 3D sequence [TR/TE: 50/2.5 ms, av: 3, TA: 2:20 h, applied flip angles (FA): FA1 = 8°, FA2 = 42°]. T2- (spin echo sequence, TR: 5,400 ms, av: 2, TA: 7:30 h) and T2^*^- (gradient echo sequence, TR: 5,400 ms, av: 3, TA: 2:00 h) relaxation time maps were acquired both on the basis of eight different echo times. Diffusion tensor imaging (DTI) was performed using a single shot echo planar imaging (EPI) sequence with b values of 0, 200, 400, 600, 800, 1,000 s/mm^2^ in 265 diffusion directions. In plane resolution of all sequences besides DTI was 67 μm^2^ with a slice thickness of 300 μm. For DTI the resolution was 300 μm isotropic. Based on morphological images, volumes of LV, striata and the corpus callosum (CC) were segmented using Chimaera's segmentation tool (Chimaera GmbH, Germany).

By transferring the segmentation mask to the respective images, T1-, T2-, and T2^*^-relaxation times, apparent diffusion coefficient (ADC) and fractional anisotropy (FA) were quantified within these volumes. The resulting parameter values were subjected to Shapiro-Wilk normality test, confirming that data were not inconsistent with a Gaussian distribution, and tested for significant differences using an unpaired *t*-test (parametric, two-tailed).

## Results

### General health screening and body weight

Longitudinal monitoring of the animals' general health status did not reveal any impairment of tg rats compared to their wt littermates. No resting tremor, ataxia, clasping, or seizures were observed. However, at later age points, transgenic animals occasionally showed opisthotonus-like movements of the head. Other behavioral abnormalities were exclusively detected in dedicated phenotyping experiments. With on average 8 pups per litter, breeding performance of F344 mothers is lower compared to the SPRD rat strain.

**Figure 1:**
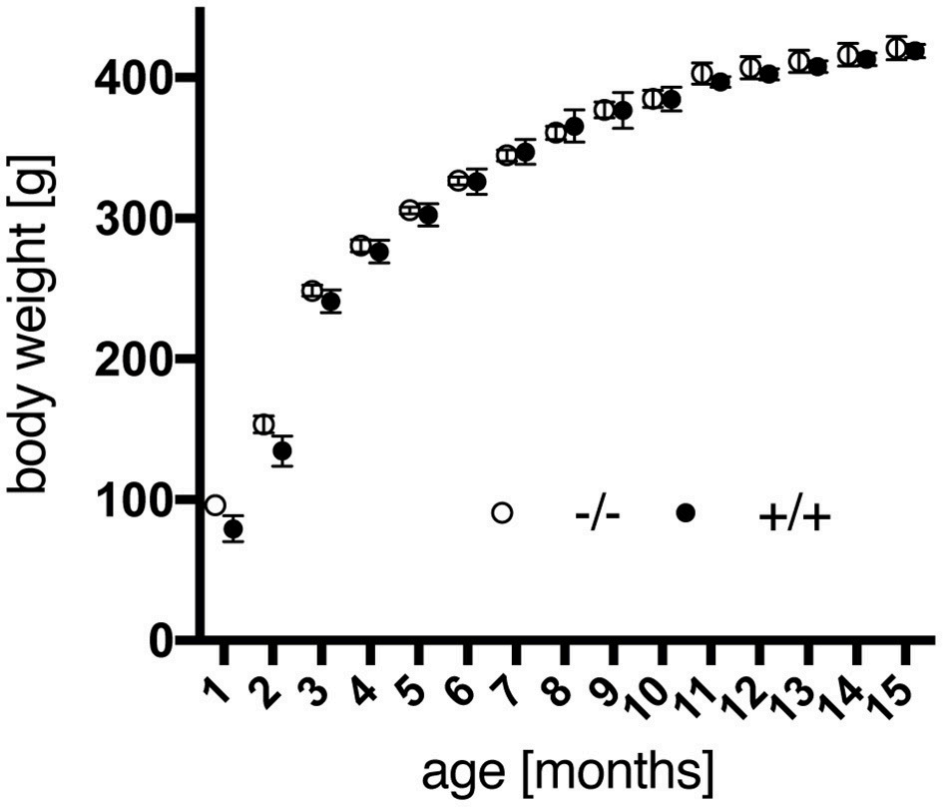
Body weight gain of male wild-type (–/–, *n* = 9) and transgenic (+/+, *n* = 11) F344 rats from 1 to 15 months of age. No significant differences were detected between both genotypes. Data are shown as mean ± SEM.

Besides, adolescent tg rats tended to weigh less than wt animals, yet over the time of investigation no significant impact of the genotype regarding weight gain [genotype, *F*_(1, 18)_ = 0.1219, *p* = 0.7310] was observed (Figure 1). More recent studies using the SPRDtgHD rats report on the same phenomenon regarding body weight (Kirch et al., 2013; Urbach et al., 2014).

### Accelerod test

**Figure 2:**
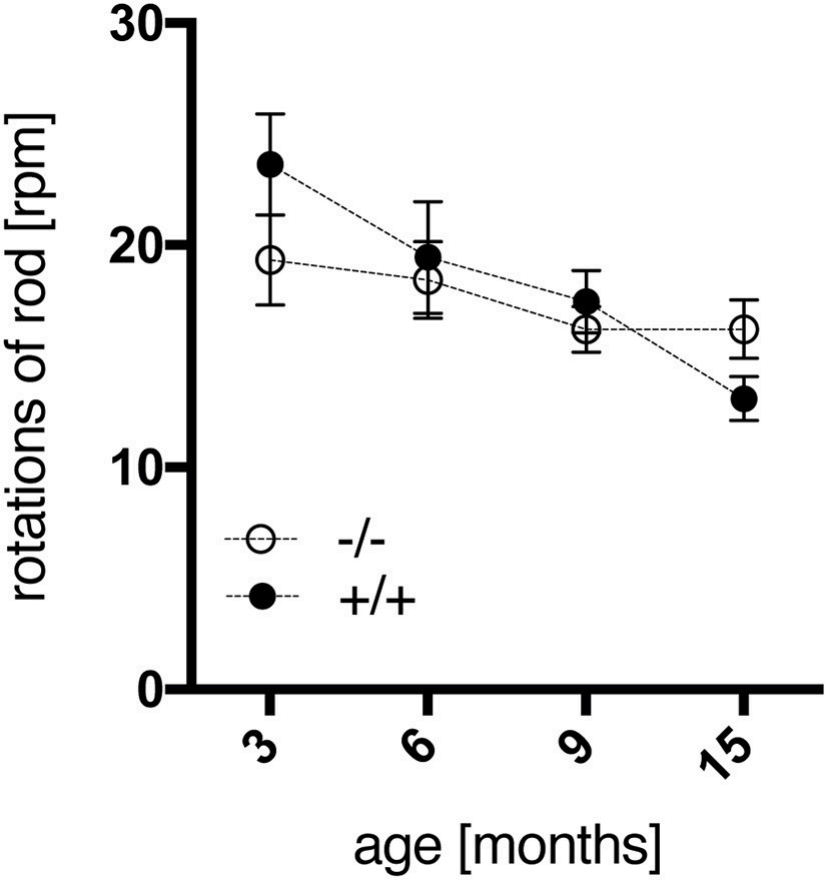
Motor coordination and balance of wt (–/–, *n* = 9) and tg (+/+, *n* = 11) rats were measured in the accelerod test at the age of 3, 6, 9, and 15 months. The mean of maximum rpm values achieved in each of two consecutive tests per age point was calculated per animal. Data are shown as mean ± SEM.

The accelerod test was used to assess the animals' motor coordination and balance. All rats reached a stable level of performance during training sessions at constant speed before testing. Surprisingly, no significant genotype-dependent differences were detected in the accelerod using two-way repeated measures Anova, with the intra-individual factor “max. rpm across age” and the inter-individual factor “genotype,” which neither depicted a significant interaction of both factors [genotype, *F*_(1, 18)_ = 0.217, *p* = 0.6469; age × genotype, *F*_(3, 54)_ = 2.248, *p* = 0.0932]. However, tg animals displayed a trend to better coordination skills at the age of 3 months, yet progressive deterioration of performance with time, in contrast to a rather consistent efficiency of wild-type rats (Figure 2). Similar findings have been reported for SPRDtgHD rats as well (Nguyen et al., 2006; Urbach et al., 2014).

### Open field test

In the open field test performed at the age of 6 months, no significant genotype-related differences could be observed (data not shown).

### Social interaction test of anxiety

**Figure 3:**
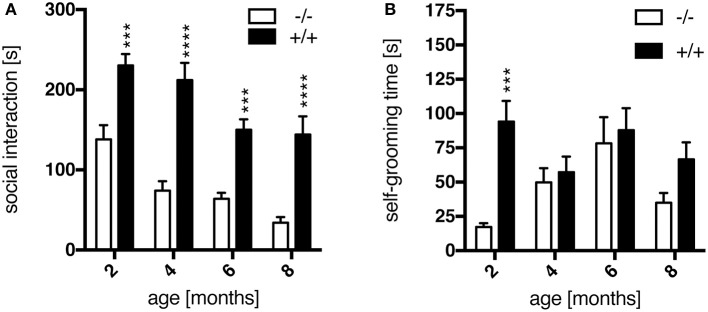
Anxiety-like behavior assessed in the social interaction test. Transgenic (+/+, *n* = 10) rats spent significantly more time in social interaction **(A)** than their wild-type (–/–, *n* = 8) littermates at all ages tested, as well as with self-grooming **(B)** at 2 months of age. Asterisks indicate significant differences between wt and tg rats (^***^p < 0.001, ^****^p < 0.0001, *post-hoc* Uncorrected Fisher's LSD). Data are shown as mean ± SEM.

The SI test was applied as a standard method to measure anxiety-related socio-positive behaviors in pairs of rats as the time spent in active social interaction in a novel environment (File and Seth, 2003). In this study, transgenic rats interacted significantly more than their wild-type littermates at all ages tested, as shown in Figure 3A [genotype, *F*_(1, 64)_ = 83.89, *p* < 0.0001]. A standard interpretation of such increased social interaction behavior is that respective experimental animals display reduced anxiety levels. *Post-hoc* testing revealed a significant genotype effect on the time spent in social interaction at each age point (Figure 3A). These results correspond to observations made in the SPRDtgHD model (Nguyen et al., 2006; Urbach et al., 2014).

Additionally, the time an animal spent for self-grooming per test session was recorded. At the age of 2 months, transgenic rats showed significantly more self-grooming than wt animals (Figure 3B), which is considered as displacement activity and probably induced by a differential perception of the novel situation. This genotype-specific difference is lost at later age points (Figure 3B), supposedly due to a habituation effect with regard to the test procedure and environment primarily in wild-type rats.

### Prepulse inhibition (PPI) of the startle response

**Figure 4:**
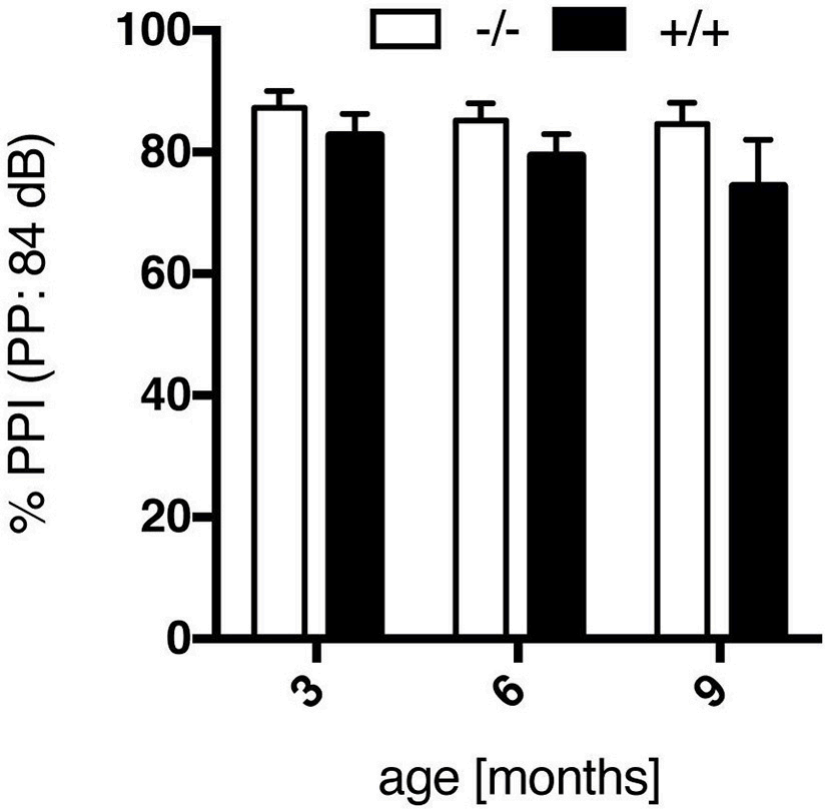
Prepulse inhibition (%), evoked by a prepulse intensity of 84 dB, of wt (–/–, *n* = 9) and tg (+/+, *n* = 11) rats at the age of 3, 6, and 9 months. Data show a trend to impairment in PPI in transgenic rats, yet not reaching significance in comparison to wt rats. Data are shown as mean ± SEM.

Prepulse inhibition of the startle response is the phenomenon by which a weak, non-startling, stimulus (prepulse) suppresses the response to a startling noise. Huntington disease patients display an impairment in PPI, which is commonly attributed to defects in cortical-striatal neuronal pathways. Here, in both wt and tg rats the intensity of the prepulse and the resultant magnitude of inhibition of the startle reaction correlated at all ages tested, with maximal inhibition at a prepulse intensity of 84 dB (Supplementary Figure 1). An age-related comparison of PPI values at this prepulse intensity indicated a trend to reduced PPI in transgenic animals (Figure 4), yet not reaching significance [genotype, *F*_(1, 18)_ = 2.177, *p* = 0.1574]. In the SPRDtgHD model, no significant, genotype-related differences in PPI were detected either (Hohn et al., 2011; Urbach et al., 2014).

### Automated intra-home-cage testing

Spontaneous locomotion, food and water intake as well as indirect calorimetry were assessed for each individual rat in a home cage-like environment in the PhenoMaster system.

#### Activity

**Figure 5:**
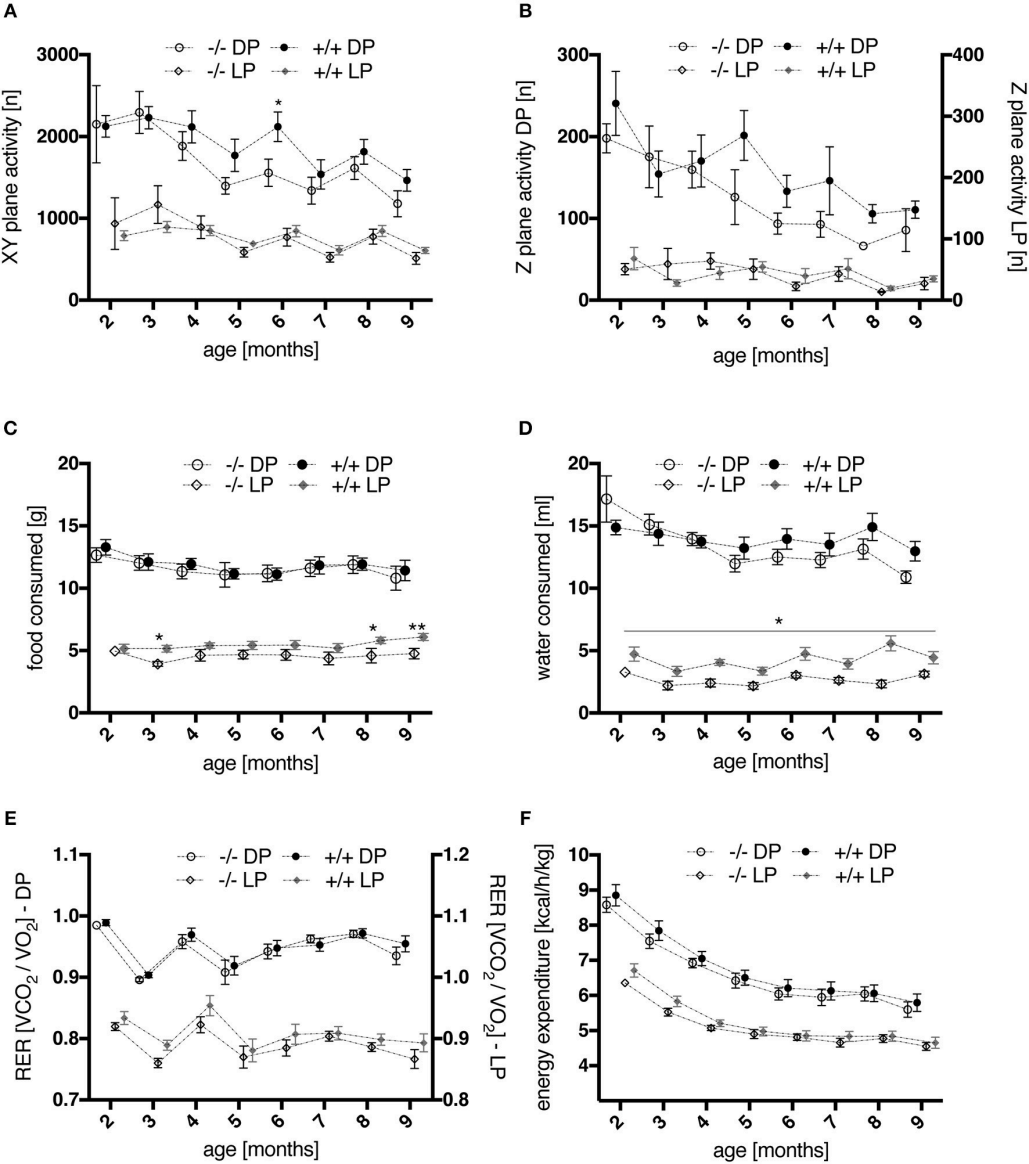
Time courses of activity and metabolic parameters, measured via automated phenotyping, during light (LP) and dark (DP) phases of wt (–/–, *n* = 9) and tg (+/+, *n* = 11) rats. **(A,B)** spontaneous home cage activity: profiles of XY-plane locomotor activity **(A)** as well as of rearing behavior **(B)** showed a progressive decline in overall activity level with advancing age. Activity levels of transgenic rats appeared elevated compared to wt controls during night phases from 4 months onwards, with a significant difference in horizontal locomotion at 6 months of age (*p* = 0.0444, *post-hoc* Uncorrected Fisher's LSD). **(C,D)** ingestion behavior: During the light phases of the daily cycle, transgenic animals consumed significantly more food at 3, 8, and 9 months **(C)** and water at all ages tested **(D)** (^*^p < 0.05, ^**^p < 0.01, *post-hoc* Uncorrected Fisher's LSD). **(E,F)** indirect calorimetry: No significant effect of genotype was detected for mean RER values **(E)** nor for energy expenditure (heat production) **(F)** regarding the overall age course. However, a moderately increased RER was observed in young tg rats compared to wt controls, most prominent at the age of 3 months **(E)**. All data are shown as mean ± SEM.

Starting at the age of 4–5 months, spontaneous activity (locomotion and rearing) in the home cage appeared to be increased in tg rats during the dark phases, when rats are mostly active (Figures 5A,B). No significant differences were detected regarding the light, i.e., resting, phases. In particular, significantly more horizontal activity was recorded at the age of 6 months in tg as compared to wt rats. In both genotypes a progressive decline in overall activity level was observed with advancing age [age, *F*_(7, 126)_ = 13.43, *p* < 0.001]. A similar activity profile has been reported for transgenic SPRDtgHD rats (Urbach et al., 2014).

#### Food and water consumption

Overall, food (Figure 5C) and water (Figure 5D) consumption in dark phases of the light cycle were similar in wt and tg rats. However, two-way repeated measures ANOVA revealed a significant effect of genotype on food [genotype, *F*_(1, 18)_ = 5.029, *p* = 0.0377] and water intake [genotype, *F*_(1, 18)_ = 17.99, *p* = 0.0005] during light phases, as well as a significant interaction of age and genotype for the latter [age × genotype, *F*_(7, 126)_ = 2.447, *p* = 0.0219]. *Post-hoc* testing confirmed significantly increased food consumption in tg rats at the age of 3, 8, and 9 months, and increased water intake at all ages studied.

#### Indirect calorimetry

In general, mean values of the respiratory exchange rate (RER) measured during the dark phases did not reveal significant differences between genotypes (Figure 5E). Two-way repeated measures ANOVA across all age points did not detect a significant effect of genotype for mean RER values regarding light phases either. However, a moderately increased RER was observed in young tg rats, most prominent at the age of 3 months, reaching significance when statistics were applied on this age point [two-way repeated measures ANOVA on factor “genotype” and factor “day phase”; genotype, *F*_(1, 18)_ = 5.495, *p* = 0.0308; light phase × genotype, *F*_(1, 18)_ = 6.31, *p* = 0.0218]. The volume of O_2_ consumed and CO_2_ produced (Supplementary Figure 2) was not significantly altered in adult transgenic rats. In young animals, only non-significantly increased values of both parameters were measured in comparison to wt controls. The same observations were made regarding energy expenditure profiles (Figure 5F).

### Operant learning

Operant conditioning is an established, valuable tool for the analysis of cognitive function in rodents, allowing for the dissection of motivational, emotional and purely cognitive aspects of learning and memory performance. The animal's behavior is conditioned via positive or negative reinforcement of deliberate actions such as lever pressing. In this study, all animals tested were successfully trained within one night to use the levers of an operant wall in order to obtain a rewarding food pellet. Subsequently, their behavioral adaptability was tested by means of dedicated operant schedules at the age of 3, 6, and 12 months.

#### Randomized alternation task

**Figure 6:**
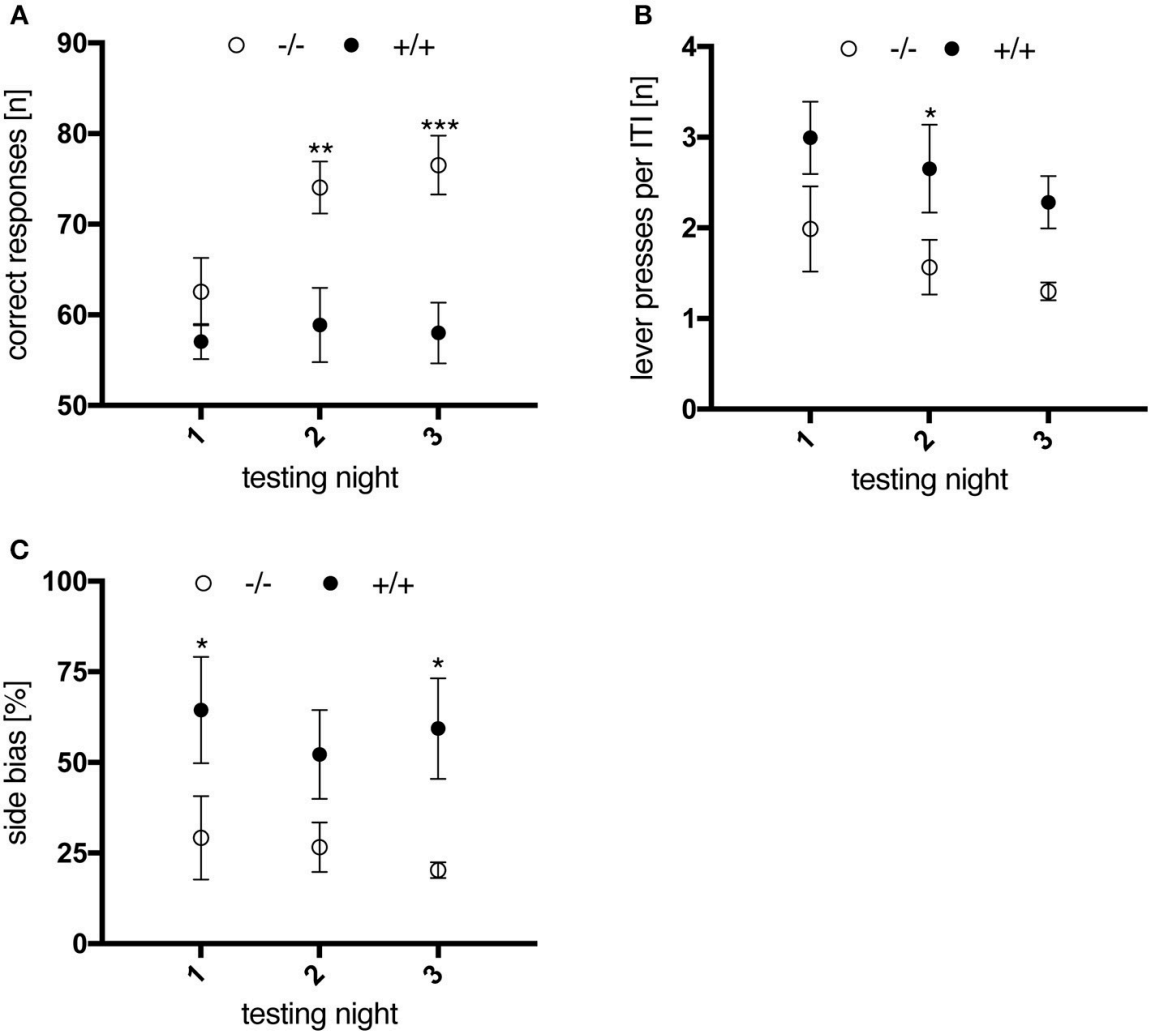
Performance of 3-month-old wt (–/–, n = 6) and tg (+/+, *n* = 6) rats in the randomized alternation task. Data are displayed as mean values of three sessions per testing night. Asterisks indicate significant differences detected by multiple comparisons (*post-hoc* Uncorrected Fisher's LSD). **(A)** Number of correct responses. Wild-type animals made significantly more progress in performing the task compared to transgenic rats (^**^p = 0.0026; ^***^p = 0.0004). **(B)** Mean number of lever presses per 10 s intertrial interval, of which transgenic animals performed more compared to wt controls (^*^p = 0.0436). **(C)** Preferential use of one lever, expressed as % side bias. Transgenic animals showed a higher tendency to use the same lever compared to wt controls (^*^p = 0.0326/*p* = 0.0189). All data are shown as mean ± SEM.

In the randomized alternation task, associative conditioning was trained by visual signaling to the rat which one of the two levers should be operated. All animals managed to complete each of three sessions per testing night within 30 min (data not shown). Compared to wild-type rats, the success rate of 3-month-old transgenic animals did not improve within three consecutive testing nights (Figure 6A); two-way repeated measures Anova revealed a significant effect of “genotype” [*F*_(1, 10)_ = 14.22, *p* = 0.0037] and “testing night” [*F*_(2, 20)_ = 4.76, *p* = 0.0204] on the animals' performance. Additionally, transgenic rats produced more lever presses during the inter-trial interval (Figure 6B; genotype, *F*_(1, 10)_ = 6.027, *p* = 0.034] and displayed a more distinct preference for one of the levers (i.e., side bias, Figure 6C) than wt controls. These results might indicate that 3-month-old tgHD rats exhibited a higher level of impulsivity, paid less attention to the light stimulus, or did not fully understand its relevance. However, the difference between wt and tg rats was not observed anymore when the same subjects were re-tested at 6 and 12 months of age (data not shown), which could be explained by further training on the light stimulus in the consecutive DRL task.

#### DRL task

Investigation of impulsivity traits continued with the DRL task, where animals were required to operate one of the levers only after a defined waiting time (inter-response time interval, IRT) to receive a reward (behavior inhibition).

**Figure 7:**
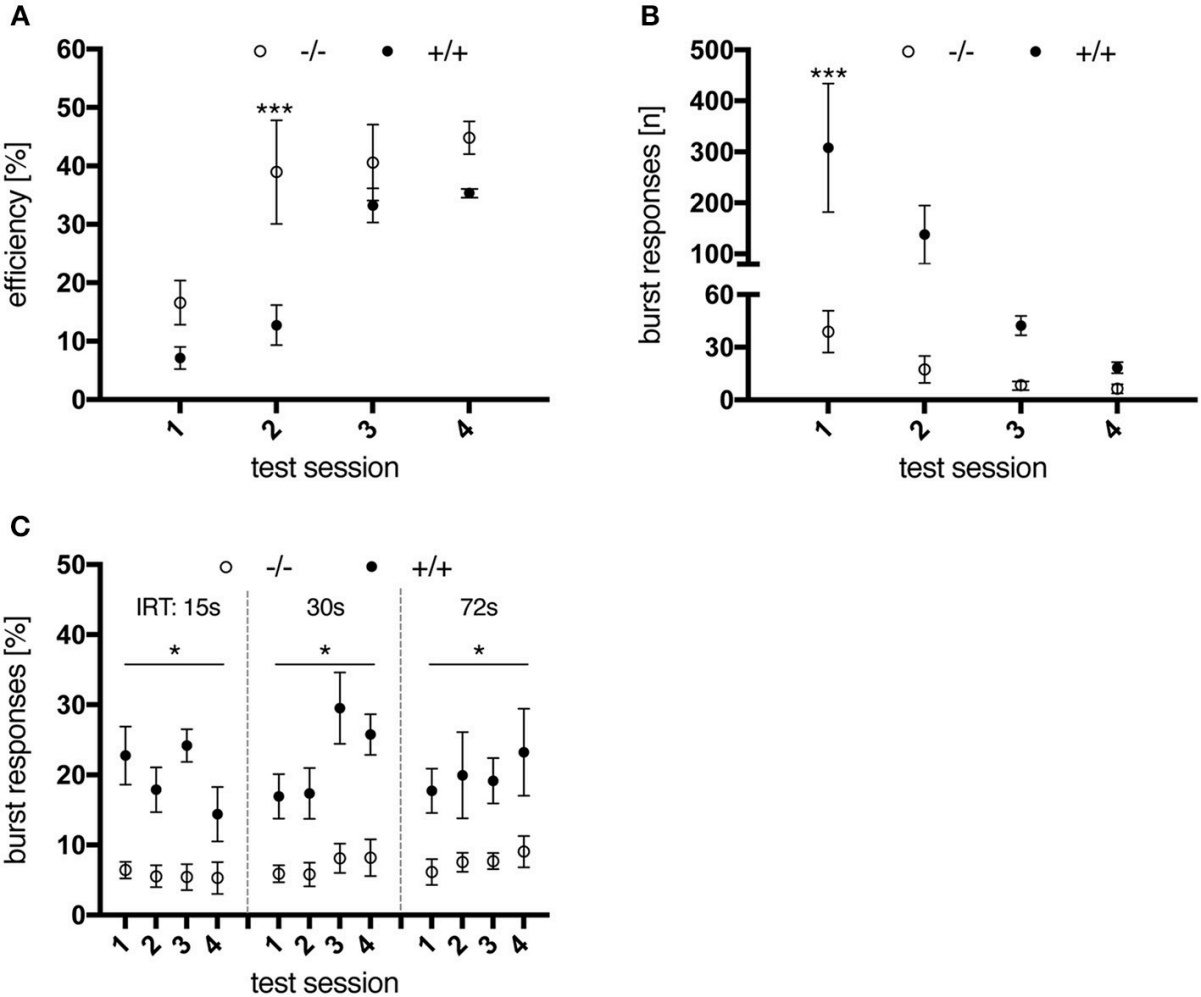
Performance of 3-month-old wt (–/–, *n* = 6) and tg (+/+, *n* = 6) rats in a DRL 15 s task. **(A)** Performance efficiency, calculated as ratio of rewarded to total responses. Wild-type animals were more efficient in performing the task due to less premature responses. **(B)** total number of burst responses (<1s) per session. Transgenic animals were significantly less able to restrain their response in the first test sessions compared to wt controls, yet improved with further testing. However, the percentage of burst responses per total premature responses **(C)** remained significantly increased for tg rats in all test sessions and for all inter-response time intervals (IRT) applied. Asterisks indicate significant differences detected by multiple comparisons (^*^p < 0.05, ^***^p < 0.001, *post-hoc* Uncorrected Fisher's LSD). All data are shown as mean ± SEM.

At an IRT of 15 s, wild-type animals performed significantly better and learned significantly quicker than their transgenic littermates [genotype, *F*_(1, 10)_ = 6.25, *p* = 0.0314; test session × genotype, *F*_(3, 30)_ = 4.062, *p* = 0.0155; Figure 7A], due to a lower frequency of premature responses. However, tgHD rats also managed to learn the task over time, as indicated by a progressive reduction in total burst responses (IRT < 1s) [Figure 7B; test session, *F*_(3, 30)_ = 5.877, *p* = 0.0028; genotype, *F*_(1, 10)_ = 5.615, *p* = 0.0393; test session × genotype, *F*_(3, 30)_ = 3.734, *p* = 0.0216]. Yet interestingly, the measured ratio of burst responses to total premature lever presses remained increased in tg rats as compared to wt littermates, in all sessions and following tests where longer IRTs were applied [Figure 7C; genotype, *F*_(1, 10)_ = 69.02, *p* < 0.0001; test session × genotype, *F*_(11, 110)_ = 0.7343, *p* = 0.7036]. Regarding performance efficiency, shifting to larger IRTs of 30 s and 72 s did not reveal any genotype-related differences, neither did a re-testing at the age of 6 months with all IRTs as applied before (data not shown). These results indicate that transgenic rats learned to perform the task as efficiently as wild-type animals at 3 months of age, but still displayed constantly elevated levels of impulsivity.

#### Progressive ratio task

Last, the animals performed the Progressive Ratio (PR) schedule, where high rates of response are encouraged and rewarded. This final test was applied in order to experimentally measure the animals' levels of motivation to work for the rewards, but also to assess their tendency to adopt stereotypical response patterns and reveal potential compulsive traits in the tg rats.

**Figure 8:**
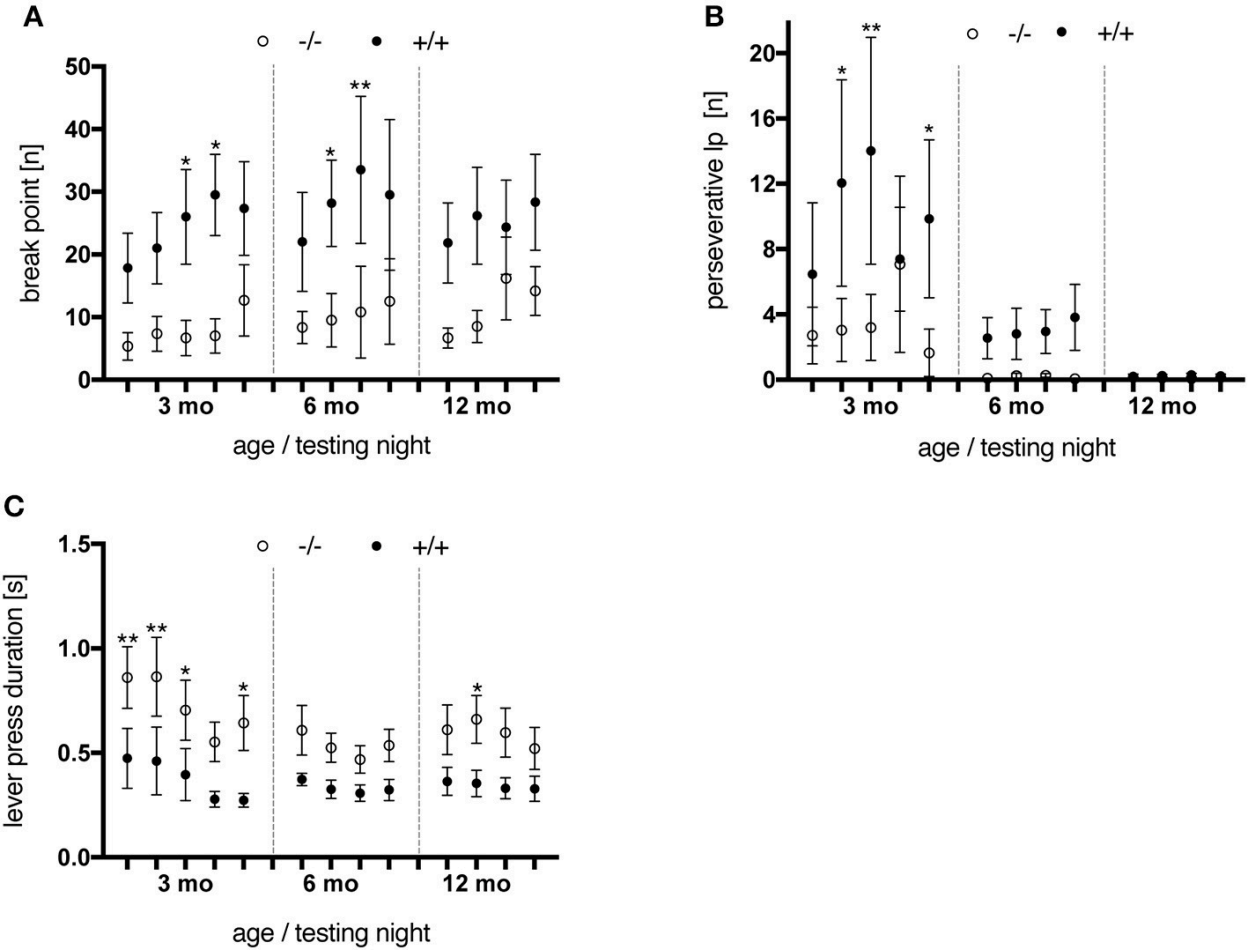
Performance of wt (–/–, *n* = 6) and tg (+/+, *n* = 6) rats on a progressive ratio schedule. Animals were tested at the age of 3 months (testing night 1–5), 6 months (night 6–9) and 12 months (night 10–13). Asterisks indicate significant differences (^*^p < 0.05, ^**^p < 0.01, *post-hoc* Uncorrected Fisher's LSD). **(A)** Break points measured per testing night. Transgenic animals reached higher break point values than wt rats at all age points tested. **(B)** Perseverative lever presses (lp) per rewards earned. Wild-type rats performed less extra lever presses before retrieving a delivered pellet compared to their tg littermates. An age-dependent decline was observed for both genotypes, yet more distinct in tg rats. **(C)** The median lever press duration of transgenic animals was shorter at all age points investigated. All data are shown as mean ± SEM.

Animals were tested on the PR schedule at the age of 3, 6, and 12 months. Transgenic rats reached higher break point values than control subjects [genotype, *F*_(1, 10)_ = 6.335, *p* = 0.0305] throughout all testing nights and age points (Figure 8A). Besides, tgHD animals performed more perseverative lever presses (i.e., lever presses produced after delivery of a food pellet) before retrieving the reward (Figure 8B), a phenomenon which decreased with age [testing night, *F*_(12, 120)_ = 3.868, *p* < 0.0001]. No significant genotype-related differences were observed regarding the total number of rewards earned per testing night (data not shown). Finally, the median duration of a lever press was significantly longer in wt animals [genotype, *F*_(1, 10)_ = 6.505, *p* = 0.0288] at all ages tested (Figure 8C). Similar results were reported for the SPRDtgHD model (Urbach et al., 2014).

Overall, regarding the break-point values as the behavioral correlate of motivation suggests that tgHD rats were more motivated in performing the PR schedule than their wt littermates. However, considering the elevated levels of perseverative lever presses observed in transgenic rats, higher break points could have also been reached due to lack of output control.

#### Acute DRL testing after PR task

**Figure 9:**
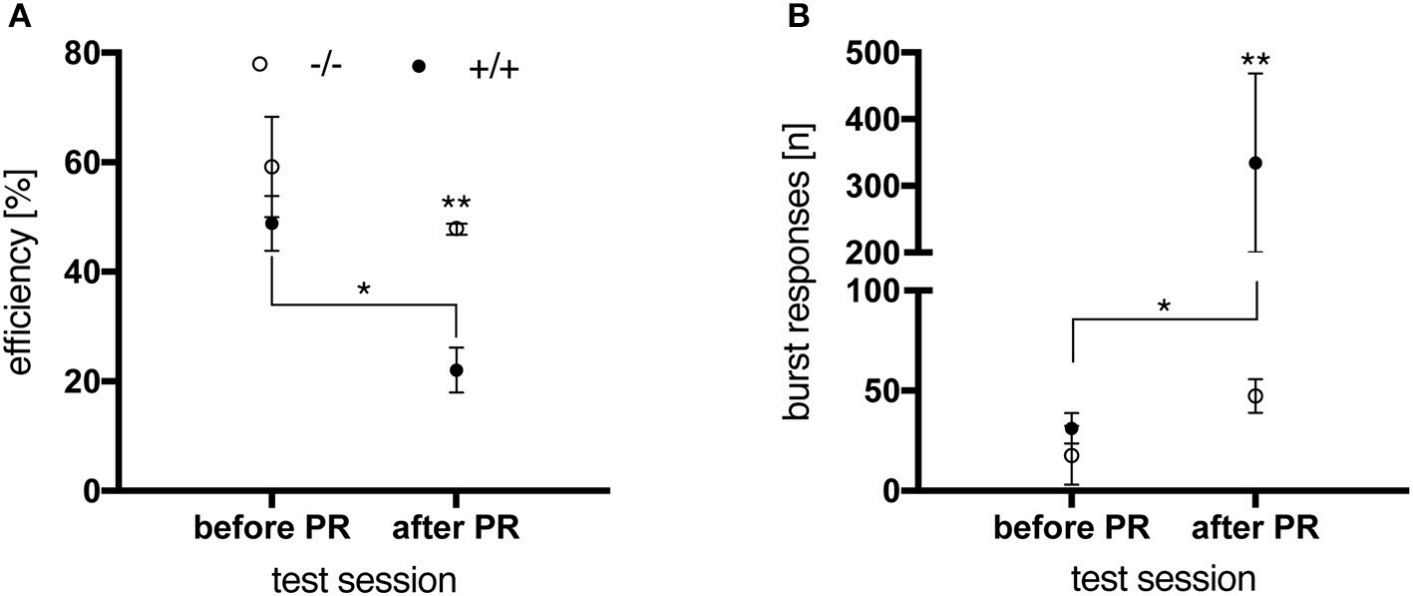
Performance of 6-month-old wt (–/–, *n* = 6) and tg (+/+, *n* = 6) rats in a DRL 15 s-task before and after PR. **(A)** After PR testing, transgenic animals were significantly less efficient in performing the task than before PR (^*^p = 0.0138) and significantly less compared to wt controls (^**^p = 0.0040). **(B)** Before PR testing, no significant difference in the total number of burst responses was observed between genotypes. Subsequent to PR training, tg rats gave significantly more burst responses than before (^*^p = 0.0126) and compared to wt animals (^**^p = 0.0071, *post-hoc* Uncorrected Fisher's LSD). All data are shown as mean ± SEM.

In order to test this hypothesis, a final DRL test with an IRT of 15 s was conducted on the night immediately following the last PR session, in animals aged 6 months. Results were compared to the animals' performance in the DRL 15 s-task preceding the PR schedule (Figure 9). While no genotype-related difference was seen in the latter regarding performance efficiency (Figure 9A) and premature burst responses (Figure 9B), preceding PR training had an impact on the transgenic animals' behavior. In contrast to control rats, which efficiently managed to switch their behavioral response between the two tasks, tgHD rats were significantly impaired in performing the acute DLR task after PR testing [Figure 9B; genotype, *F*_(1, 10)_ = 5.399, *p* = 0.0425; time, *F*_(1, 10)_ = 5.546, *p* = 0.0403] resulting in a significantly reduced performance efficiency [Figure 9A; genotype, *F*_(1, 10)_ = 14.25, *p* = 0.0036; time, F_(1, 10)_ = 8.959, *p* = 0.0135].

Altogether, these data substantiate the impulsive/compulsive phenotype of tgHD rats displayed in operant conditioning tasks, which has been reported for older SPRDtgHD rats by El Massioui et al. (2016).

### Immunohistochemistry

**Figure 10:**
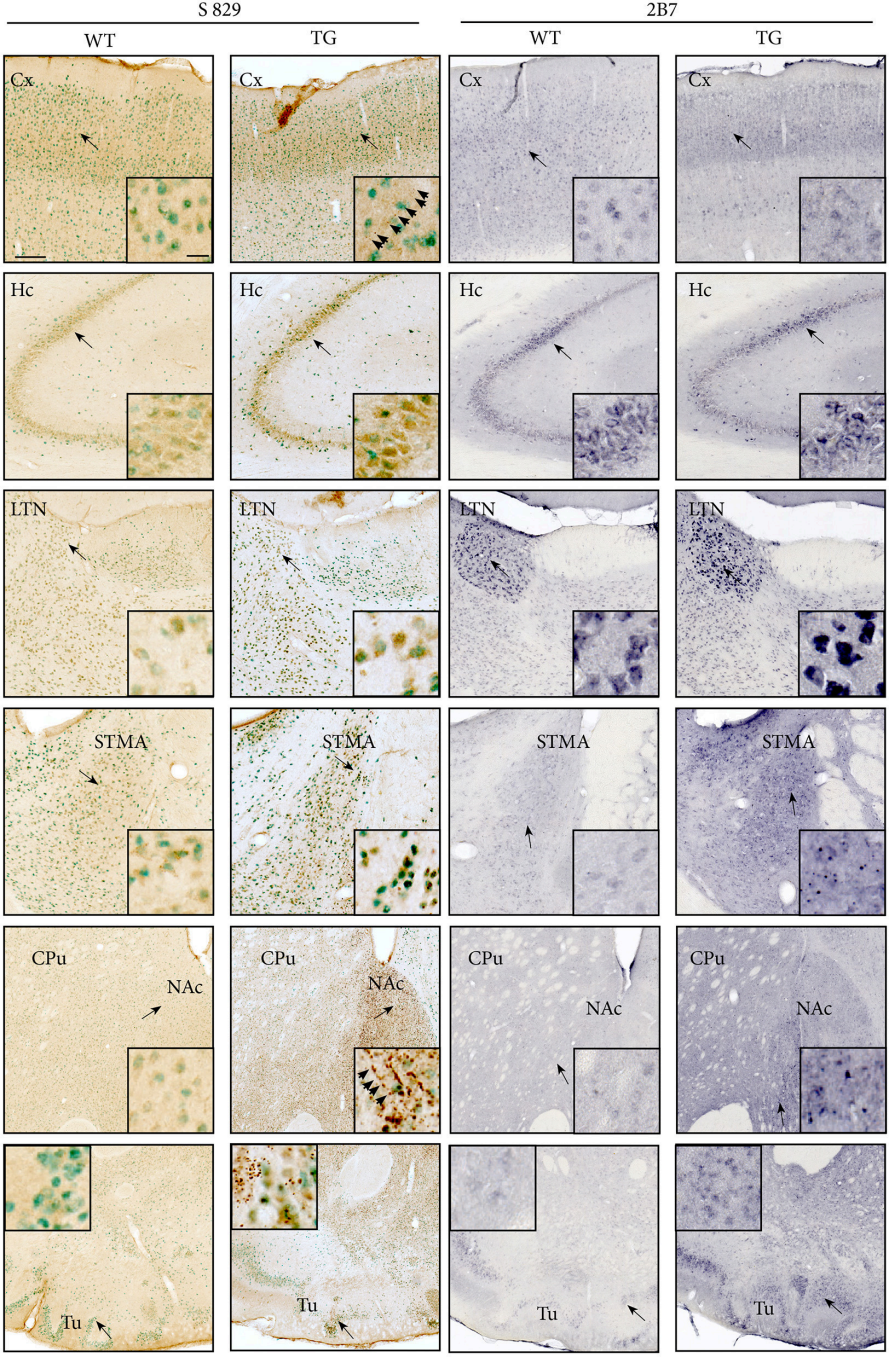
The distribution of mHTT and HTT in brains of 21-month-old F344tgHD tg and wt rats was analyzed using polyclonal sheep antibody S829 (visualized with DAB, seen as brown deposit; NeuN-immunoreactivity seen as green) or monoclonal mouse antibody 2B7 (visualized with Nickel-DAB). Rows show images from selected brain regions: Somatosensory cortex (Cx, row 1), CA2/CA3 area of the hippocampus (Hc, row 2), laterodorsal thalamic nucleus, dorsomedial part (LTN, row 3), bed nucleus of stria terminalis (STMA, row 4), striatum and nucleus accumbens (CPu/NAc, row 5), and piriform cortex and olfactory tubercle (Tu, row 6). Black arrows indicate the site of the inset image. S829 mHTT immunoreactivity was observed throughout the cerebrum of tg rats, most abundantly in the nucleus accumbens and olfactory tubercle. Immunoreactivity for 2B7 HTT was clearly identified in several regions, particularly strong in the laterodorsal thalamic nucleus, dorsomedial part (LTN, row 3). Aggregates of varying size were mainly localized in the neuropil, and partly arranged in a linear, chain-like array (arrowheads). Scale bars: low-magnification images: 200 μm; inset showing high-magnification images: 20 μm.

**Figure 11:**
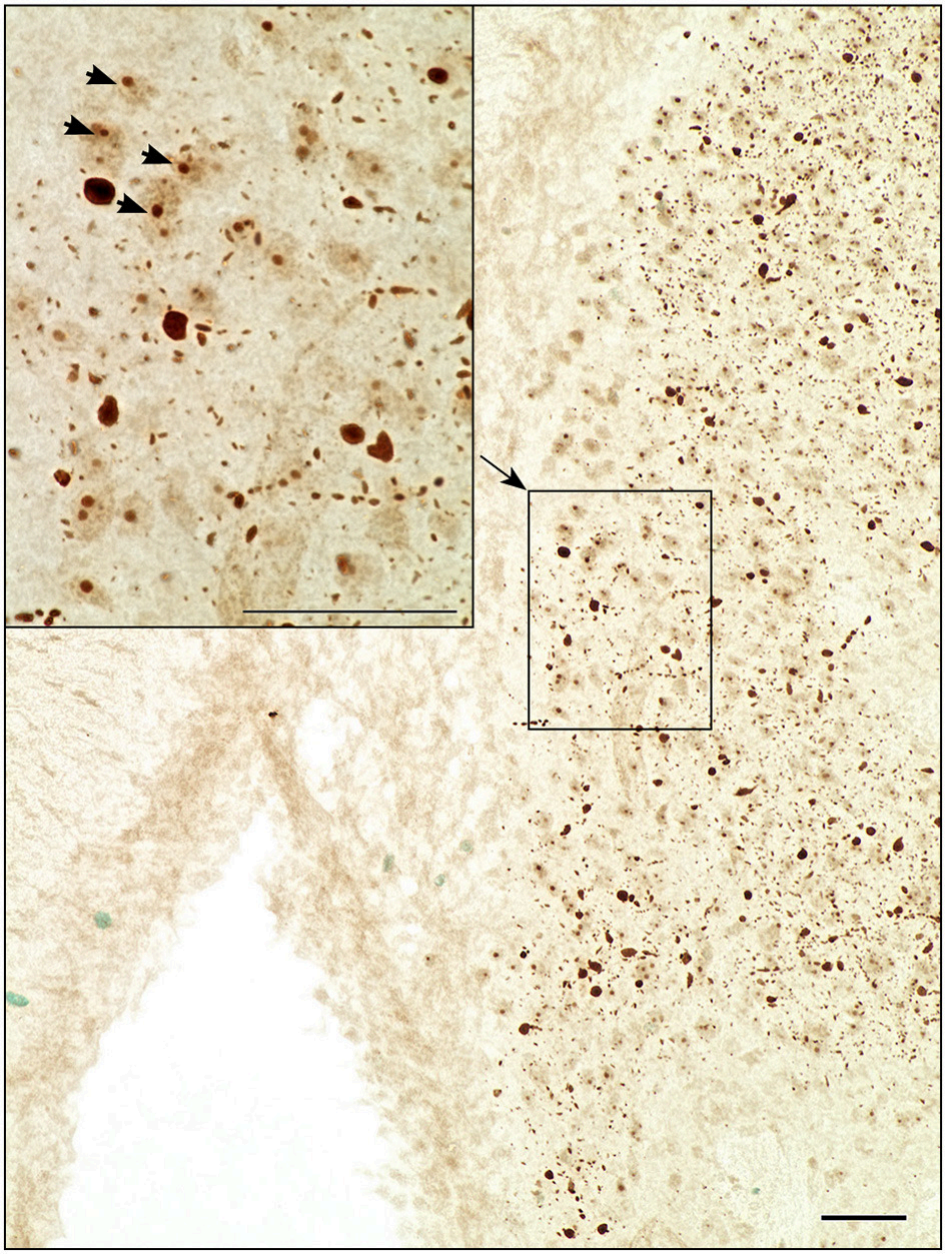
Light microscopic investigation of the striatum at higher magnification revealed nuclear localization of S829 immunoreactivity (arrowheads) as well as chain-like arrangements of aggregates in the neuropil. Scale bars: 50 μm.

Distribution patterns of immunohistochemically stained HTT and mHTT-positive aggregates were investigated in serial sections through the brain. Immunoreactivity of the polyclonal sheep antibody S829 and the monoclonal mouse antibody 2B7 on tissue from 21-month-old female wt and tg rats is shown in Figure 10. The 2B7 antibody detects both wild-type and mutated HTT, whereas S829 displays immunoreactivity with mHTT aggregates only. In tg rat brains, aggregates of varying size were found in several regions, most prominently in the bed nucleus of the stria terminalis, the nucleus accumbens and the piriform cortex and olfactory tubercle (Figure 10 rows 4, 5, and 6). Abundance of aggregates was less in the caudate putamen (Figure 10 row 5) and the neocortex (Figure 10, row 1), and low in the CA2/CA3 area of the hippocampus (Figure 10A, row 2). Most aggregates were detected in the neuropil, some of which arranged in a linear array. Nuclear localization of mHTT in the striatum is depicted in Figure 11. CNS tissue of wild-type animals did not exhibit any specific S829 immunoreactivity. Additionally, in the dorsomedial part of the laterodorsal thalamic nucleus strong immunoreactivity of 2B7 was observed in both genotypes, yet more pronounced in tg rats (Figure 10, row 3). A similar distribution of S829 immunoreactivity was detected in brain sections of 15-month-old male tg rats (Supplementary Figure 3). It has to be noted here that these sections were derived from formalin-fixed brains originally dedicated to *ex vivo* imaging studies.

### *Ex vivo* MR imaging studies

**Figure 12:**
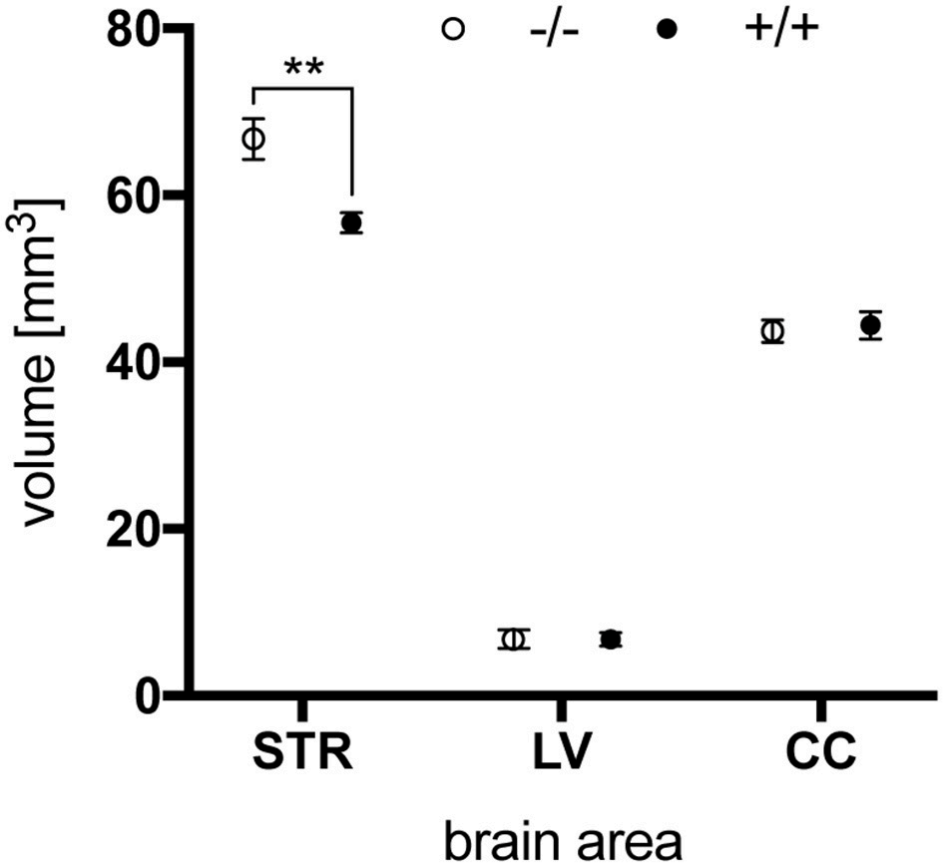
Total volume of striatum (STR), lateral ventricle (LV) and corpus callosum (CC) of 15-month-old wt (–/–, *n* = 6) and tg (+/+, *n* = 6) rats. Striatal volume was significantly reduced in tg rat brains (^**^p = 0.0042, unpaired *t*-test) while no differences were seen in the size of lateral ventricles and corpus callosum. Data are shown as mean ± SEM.

Finally*, ex vivo* imaging studies were conducted on brains of 15-month-old animals to examine potential morphologic alterations in tg rats compared to wt controls. Brains were scanned on a preclinical ultra-high field magnetic resonance imaging system, and volumes of LV, striata (STR), and CC were determined based on morphologic images obtained with a T1-weighted turboflash magnetization-prepared rapid gradient echo sequence. While no genotype-related differences in the size of LV and CC were detected by this technique, a significantly lower striatal volume of tgHD rat brains compared to wt controls was revealed (Figure 12). Such striatal volume reduction has been reported for 12-month-old SPRDtgHD rats (Kantor et al., 2006; Nguyen et al., 2006).

Furthermore, T1-, T2-, and T2*-relaxation times, ADC and FA were quantified within these volumes. However, no significant differences regarding genotype were observed for these parameters (Supplementary Figure 4).

## Discussion

With the objective of optimizing a formerly developed rat model of HD (SPRDtgHD), a new congenic line of the model was created by cross-breeding the transgene in the F344 strain.

Our study describes a first characterization of the behavioral and neuropathological phenotype of the newly developed F344tgHD transgenic rat model. We detected early alterations in operant performance as well as prominent huntingtin aggregation in the CNS, and substantiated several relevant phenotypic characteristics reported on the original Sprague Dawley model. A comparative summary of findings reported on both models is given in Table 1.

**Table 1:** Summary of the behavioral and neuropathological phenotype of the F344tgHD model presented in this study, in comparison to findings reported on the SPRDtgHD model.

**Parameter investigated**	**Findings in F344tgHD**	**Age of onset (months)**	**Findings in SPRDtgHD**	**Age of onset (months)**	**References (selection)**
Body weight	No differences	–	No differences	–	Kirch et al., 2013; Urbach et al., 2014
Motor function	No significant differences	–	Impaired performance in accelerod	6	Nguyen et al., 2006; Bode et al., 2008
Anxiety	Reduced in SI test	2	Reduced in SI test/Elevated plus maze	2	von Hörsten et al., 2003; Nguyen et al., 2006; Bode et al., 2008
PPI	No differences	–	No differences	−	Hohn et al., 2011; Urbach et al., 2014
Home cage nocturnal activity	Increased, yet significant differences only at 6 months	4	Increased activity	24	Bode et al., 2008; Urbach et al., 2014
Food/water consumption	increased during light phase; water: all age points tested; food: at 3, 8, and 9 months	2–3	No differences	–	Zeef et al., 2012b; Urbach et al., 2014
			Increased relative food uptake	2	Bode et al., 2008
Calorimetry	No significant differences	–	RER reduced during light phase, nocturnal energy expenditure increased	3	Urbach et al., 2014
Operant learning	Elevated impulsivity traits	3	Elevated impulsivity traits^a^	15^*^	El Massioui et al., 2016
mHTT aggregation	Prominent aggregates in basal ganglia and other brain regions^a^	21^*^	Prominent aggregates in basal ganglia and other brain regions^a^	6	Nguyen et al., 2006

a *Study was performed using female rats*.

* *Earliest time point investigated*.

Transgenic animals did not exhibit any obvious impairment in general health screening, which is a prerequisite for unbiased behavioral testing. Male homozygous F344tgHD rats did not display significant differences in body weight gain compared to wild-type littermates, similar to observations reported on the SPRDtgHD model (Kirch et al., 2013; Urbach et al., 2014). In general, 12-month-old male F344 rats of both genotypes were about 100 g lighter than Sprague Dawley wild-type animals of the same age. This is an obvious advantage of the new model with regard to certain investigations such as *in vivo* imaging. We could also rule out any confounding influence of body weight on the accelerod performance, where a deterioration was observed in transgenic animals with advancing age. However, compared to wild-type rats, a significant HD-like motor impairment was not detected, so alternative tests investigating forelimb and hind limb coordination will have to be conducted in this model. For instance, the beam walking test delivered clear genotype-related deficits in SPRDtgHD rats, starting at the age of 8 months (Nguyen et al., 2006). Ortiz and coworkers (Ortiz et al., 2012) demonstrated gait disturbances in 12- to 15-month-old tgHD rats using a force-sensing runway, and gait analysis with the Catwalk System (Noldus IT, Netherlands) revealed abnormalities in BACHD transgenic rats (Abada et al., 2013). The Catwalk System is used in ongoing studies on the F344tgHD model in our lab and could reveal motoric impairments of tg animals more robustly. Besides, motor dysfunction might still become overt on the accelerod later in life. Consistent with findings in SPRDtgHD homozygous rats (Nguyen et al., 2006; Urbach et al., 2014), young F344tgHD tg animals displayed a trend to better coordination skills than wild-type controls at the age of 3 months in this test. Early repair mechanisms leading to “hypercompensation” were discussed as potential reason for the more pronounced motoric skills at young age by Nguyen et al. with regard to the SPRDtgHD line (Nguyen et al., 2006). Such mechanisms were moreover speculated to account for the early appearance of an anxiolytic-like phenotype that we also observed in F344tgHD transgenic animals in the SI test, at all age points investigated. Our observation of a significantly increased self-grooming behavior in 2-month-old tg rats, which we consider as displacement activity probably induced by a differential perception of the novel situation, might also relate to such processes. Overall, several studies report on reduced anxiety-like behavior in SPRDtgHD transgenic animals (von Hörsten et al., 2003; Nguyen et al., 2006; Bode et al., 2008; Zeef et al., 2012b; Kirch et al., 2013), and our findings presented here confirm this emotional disturbance, representing a key symptom of HD pathology, in the F344tgHD model. Interestingly, in 6-month-old SPRDtgHD rats, Zeef and coworkers (Zeef et al., 2012b) found a strong correlation between reduced anxiety levels and hyperactivity measured as total distance moved in the open field. At this age, F344 tg rats also display significantly increased home cage activity as measured in the PhenoMaster System during their active phase, in parallel to PhenoMaster data gathered from the Sprague Dawley model (Bode et al., 2008; Urbach et al., 2014). Zeef and coworkers hypothesized that hyperkinesia could be due to a change in dopamine homeostasis in the HD brain, which might also account for a lowered anxiety-like behavior (Zeef et al., 2012b). Additionally, such dopaminergic imbalance could relate to increased impulsivity (Zeef et al., 2012b), a symptom observed in HD patients in the context of a dysexecutive syndrome (Bates et al., 2002). In the operant tasks applied in our study, impulsivity traits as well as behavioral inflexibility could be observed in transgenic rats already at 3 months of age. The DRL schedule was applied to assess the animals' ability to withhold behavioral responses for a defined time interval, in order to obtain a reward. Transgenic animals performed the DRL 15 s task less efficiently in the first test sessions than their wild-type littermates. This lower performance efficiency was due to an increased number of premature responses, including significantly more immediate burst responses, and is indicative of a lack of behavioral output control. El Massioui and coworkers report similar findings on 15-month-old female SPRDtgHD rats, with transgenic animals displaying reduced efficiency as well as increased numbers of premature and burst responses in a DRL 5 s task (El Massioui et al., 2016). However, in contrast to their findings, the efficiency of our transgenic animals increased with advancing training. On the one hand, this phenomenon might be due to the fact that we used a different DRL protocol, applying light cues that indicate the IRT frame and support associative conditioning. On the other hand, our animals were much younger and consequently in a pre-symptomatic stage of disease. These considerations could also explain why neither successive IRT-shifts to 30 and 72 s nor re-testing at the age of 6 months revealed any genotype-related differences. Yet although transgenic animals managed to improve their performance efficiency over time, their relative number of burst responses remained elevated compared to wild-types, which is clearly indicative of persisting impulsive action.

Further evidence of an impulsive phenotype was provided by data obtained in the progressive ratio task, where tgHD animals executed more perseverative lever presses and displayed a shorter lever press duration, potentially suggesting a lack of output control. Moreover, a subsequent task shift to DRL 15 s resulted in a significantly reduced performance efficiency in 6-month-old transgenic rats compared to control animals. The latter managed to directly switch between the two tasks, demonstrating behavioral flexibility that was obviously impaired in the transgenic animals. Such inflexibility in changing behavior is seen in HD patients as well, along with deficits in attention (Bates et al., 2002). Consistent with these observations, the ability of our transgenic rats to follow a light stimulus appeared to be impaired in the randomized alternation task. Instead, they preferred to continuously operate one of the two levers and produced more perseverative responses, both indicative of a rather stereotypic behavior along with lowered associative adaptability. Finally, the higher level of break point reached in the progressive ratio schedule might be due to increased motivation of transgenic rats compared to wild-type animals; yet these results could arise from a compulsive behavioral trait and lack of output control as well. In general, regarding the interpretation of all operant task data, it has to be kept in mind that animals are food restricted during testing periods. Similar to findings in the SPRDtgHD model (Bode et al., 2008), our transgenic rats' food intake was increased during day phases at 3 months of age as measured by the PhenoMaster System. This phenomenon might arise from disturbances in circadian rhythm which are too subtle to cause a significant increase in locomotion, yet could also be caused by differential metabolic conditions in tg rats. Such notion is also in line with an increased light phase RER value observed at 3 months of age. Consequently, we cannot completely rule out an impact of metabolic differences on the animals' performance in food-rewarded tests, an issue that has been discussed in detail for the BACHD rat model (Clemensson et al., 2017). However, we did not observe significant metabolic differences in 6-month-old F344tgHD transgenic rats compared to wild-type controls, while impulsivity traits were still present in the progressive ratio and consecutive DRL task, so we conclude that our transgenic rats display an early impairment of inhibitory control not likely to be confounded by differential effects of food restriction. In the Sprague Dawley line of the tgHD model, several studies report on such executive function impairments (Cao et al., 2006; Kantor et al., 2006; Hohn et al., 2011; El Massioui et al., 2016) as well as on memory deficits from 6 months of age onwards (Nguyen et al., 2006; Zeef et al., 2012a; Kirch et al., 2013). The qualitative, quantitative and temporal occurrence of the latter remains to be investigated in the F344tgHD congenic line. In this report, we demonstrate that neuropathological correlates of behavioral symptoms are pronouncedly detected in transgenic rats at advanced disease stage. For instance, *ex vivo* imaging studies on brains of 15-month-old animals revealed a significantly lower striatal volume of tg rat brains compared to wt controls, reflecting the situation seen in human pathology (Bates et al., 2002). We were not able to detect genotype-related differences in lateral ventricle size, yet this outcome might be attributed to tissue preparation processes. Volumetric analysis using a different technique could therefore display enlargement of LV, as reported for the SPRDtgHD line (von Hörsten et al., 2003; Kantor et al., 2006; Petrasch-Parwez et al., 2007). Finally, we sought to examine the distribution pattern and characteristics of mHTT-positive aggregates, a hallmark symptom of HD, in the brains of our transgenic rats. In 21-month-old animals we found aggregates of varying size and formation in several brain regions, as illustrated by S829-immunoreactivity. Though we do not have direct head-to-head comparison to the SPRDtgHD line, aggregates appeared to be markedly more abundant in the F344 transgenic rat brain at such advanced age. The cortex, nucleus accumbens, bed nucleus of the stria terminalis, piriform cortex and olfactory tubercle displayed high abundance of mutant huntingtin aggregates. These observations reflect the conditions of advanced human HD pathology (Gutekunst et al., 1999), and future studies will further characterize the regional and subcellular distribution of mHTT aggregates in this model. It should be noted here that complete whole-brain serial sections of female rats were analyzed in this study due to distinct tissue preparation applied in males; yet similar staining patterns were observed in selected brain sections of male transgenic rat tissue (Supplementary Figure 3). Still, the importance of studying gender-related phenotypic differences has been demonstrated by Bode et al. (2008) regarding the SPRDtgHD model and will have to be investigated in the F344tgHD line as well. Additionally, a detailed temporal investigation of neuropathological markers, taking into account pre-symptomatic age points, will allow for an association to the behavioral phenotype of F344tgHD rats reported in this study. Since already 3-month-old transgenic rats presented behavioral abnormalities that potentially base on altered dopamine homeostasis in HD—reduced anxiety-like behavior, impulsivity—(Zeef et al., 2012b; El Massioui et al., 2016), such investigations should include a thorough analysis of the dopaminergic system.

In summary, the novel congenic F344tgHD line of the SPRDtgHD model presents an early behavioral phenotype, comprising markedly reduced anxiety-like behavior and elevated impulsivity traits already at 3 months of age. Adult F344tgHD rats exhibit pronounced aggregation of mutated huntingtin in various brain regions and significantly reduced striatal volume. Consequently, the F344tgHD model reproduces key symptoms of HD pathology, and these results are substantiated by their analogy to findings published on the SPRDtgHD line. Compared to the latter, we assume the congenic F344tgHD line to be advantageous in terms of reproducibility of results, as well as practical usability based on their lower body weight. Altogether, we consider the F344tgHD rat as valuable model for preclinical therapeutic studies, and expect future investigations of its neuropathological and behavioral phenotype to further confirm this conclusion.

## Author contributions

A-CP and FC: conception and conduction of experiments, analyses, writing of the article. KR and YU: conception and conduction of behavioral experiments. JD, MP, and JB: conduction and analyses of histological staining, discussions. CG and TB: conduction and analyses of *ex vivo* magnetic resonance imaging. OR, HN, and SvH: creation of the rat model, conception of experiments, discussions.

### Conflict of interest statement

The authors declare that the research was conducted in the absence of any commercial or financial relationships that could be construed as a potential conflict of interest.

## Abbreviations

ADC: apparent diffusion coefficient
CC: corpus callosum
CPu: caudate putamen
Cx: cerebral cortex
FA: fractional anisotropy
F344tgHD rat: Fisher transgenic Huntington disease rat
Hc: hippocampus
HD: Huntington disease
HTT: huntingtin
mHTT: mutant huntingtin
IHC: immunohistochemistry
IRT: inter-response time interval
LV: lateral ventricles
LTN: laterodorsal thalamic nucleus
MRI: magnetic resonance imaging
NAc: nucleus accumbens
OFT: open field test
PM: PhenoMaster
PPI: Prepulse inhibition
RER: Respiratory exchange rate
SD: Sprague Dawley
SI test: Social Interaction test of anxiety
SPRDtgHD: Sprague Dawley transgenic Huntington's disease rat
STMA: bed nucleus of stria terminalis
STR: striatum
tg: transgenic
Tu: olfactory tubercle
wt: wild-type.
