# Supplementary material for: Early Alterations in Operant Performance and Prominent Huntingtin Aggregation in a Congenic F344 Rat Line of the Classical CAGn51trunc Model of Huntington Disease
Source: Front Neurosci. 2018 Jan 25;12:11. doi: 10.3389/fnins.2018.00011 (PMC5788972; doi:10.3389/fnins.2018.00011)
Supplement: Supplementary file 1 [file DataSheet1.PDF]

Supplementary Material

Early Alterations in Operant Performance and Prominent Huntingtin Aggregation in a Congenic F344 Rat Line of the Classical $CAG_{n51trunc}$ Model of Huntington Disease

Anne-Christine Plank[1], Fabio Canneva[1], Kerstin Raber[1], Julia Dobner[1], Yvonne K. Urbach[1], Maja Puchades[2], Jan G. Bjaalie[2], Clarissa Gillmann[3], Tobias Bäuerle[3], Olaf Riess[4], Huu Phuc Nguyen[4], and Stephan von Hörsten[1*]

[*]Correspondence:

Prof. Dr. Stephan von Hörsten, Experimental Therapy, Preclinical Experimental Center, University Clinics Erlangen, Friedrich-Alexander-Universität Erlangen-Nürnberg (FAU), Palmsanlage 5, 91054 Erlangen, Germany. E-mail: stephan.v.hoersten@fau.de . Tel. +499131/85-23504

Supplementary Figures

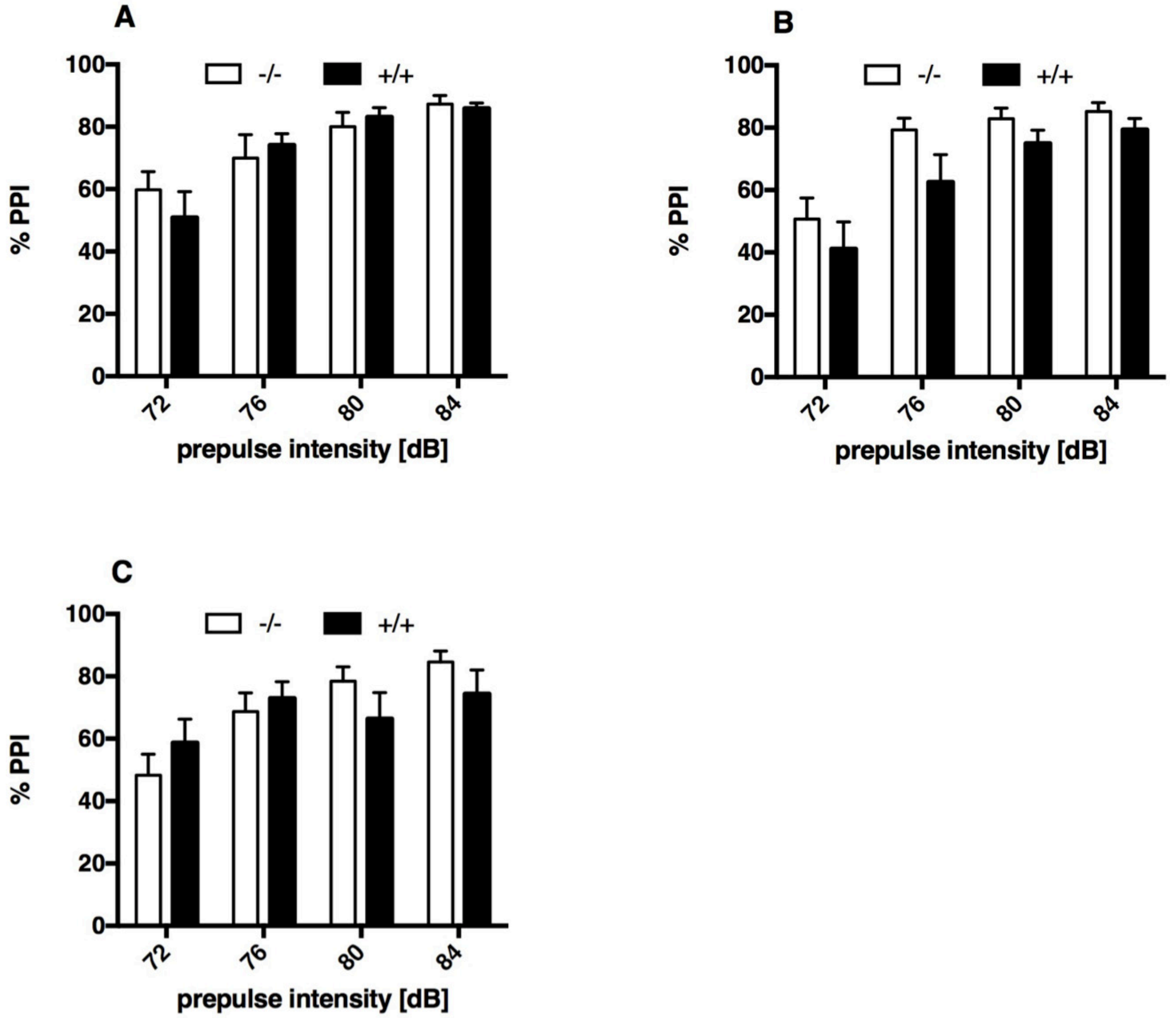

Supplementary Figure 1. Prepulse inhibition (%), evoked by prepulse intensities of 72, 76, 80 and 84 dB, of wt (-/-) and tg (+/+) rats at the age of 3 (**A**), 6 (**B**) and 9 (**C**) months. In both wt and tg rats the intensity of the prepulse and the resultant magnitude of inhibition of the startle reaction correlated at all ages tested, with maximal inhibition at a prepulse intensity of 84dB. Data are shown as mean ± SEM.

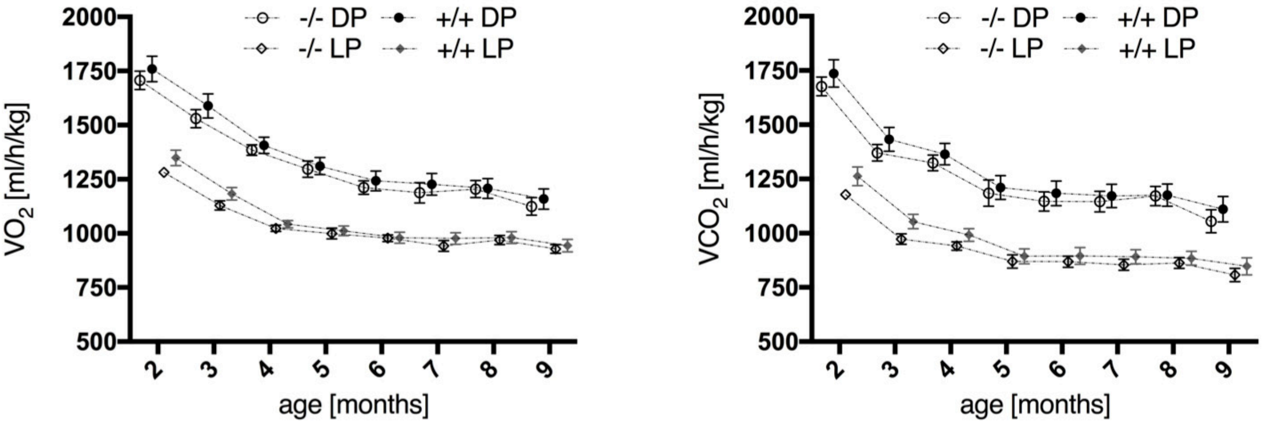

Supplementary Figure 2. Indirect calorimetry as measured by the PhenoMaster system at the age of 2 to 9 months. No significant differences in the volume of O_2 consumed and CO_2 produced were detected in transgenic animals compared to wt controls. All data are shown as mean ± SEM.

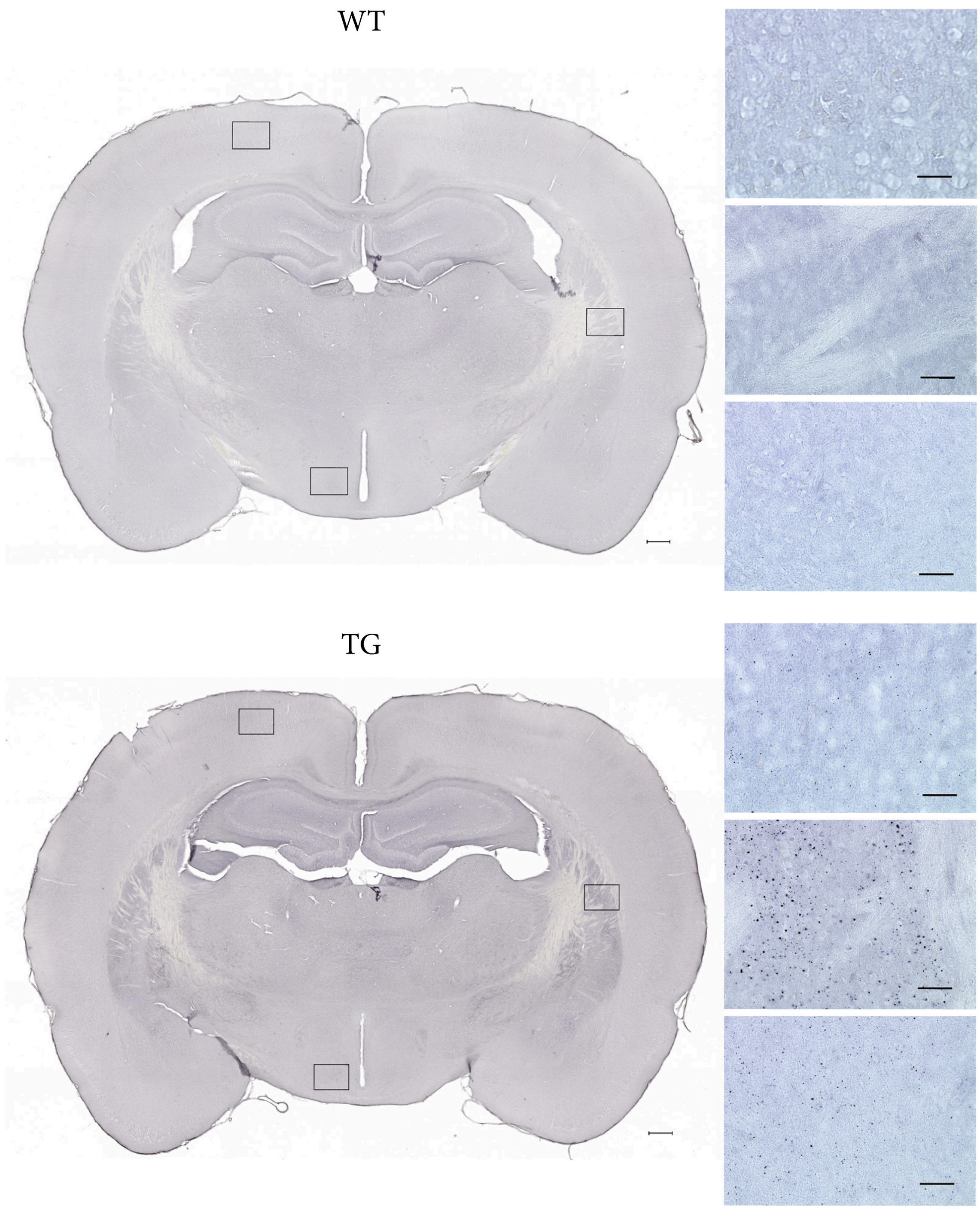

Supplementary Figure 3. Brain sections of 15-month-old male F344tgHD wt (upper panel) and tg (lower panel) rats were analyzed for S829 mHTT immunoreactivity (visualized with Nickel-DAB). Insets in overview pictures indicate the cortical, striatal and hypothalamic sites displayed in higher magnification images. Aggregates of varying size were observed throughout the brain of tg rats, with prominent aggregation in caudate putamen. Of note: tissue was derived from formalin-fixed brains originally dedicated to *ex vivo* imaging studies. Scale bars: overview images: 500 μm; insets showing higher magnification images: 50 μm.

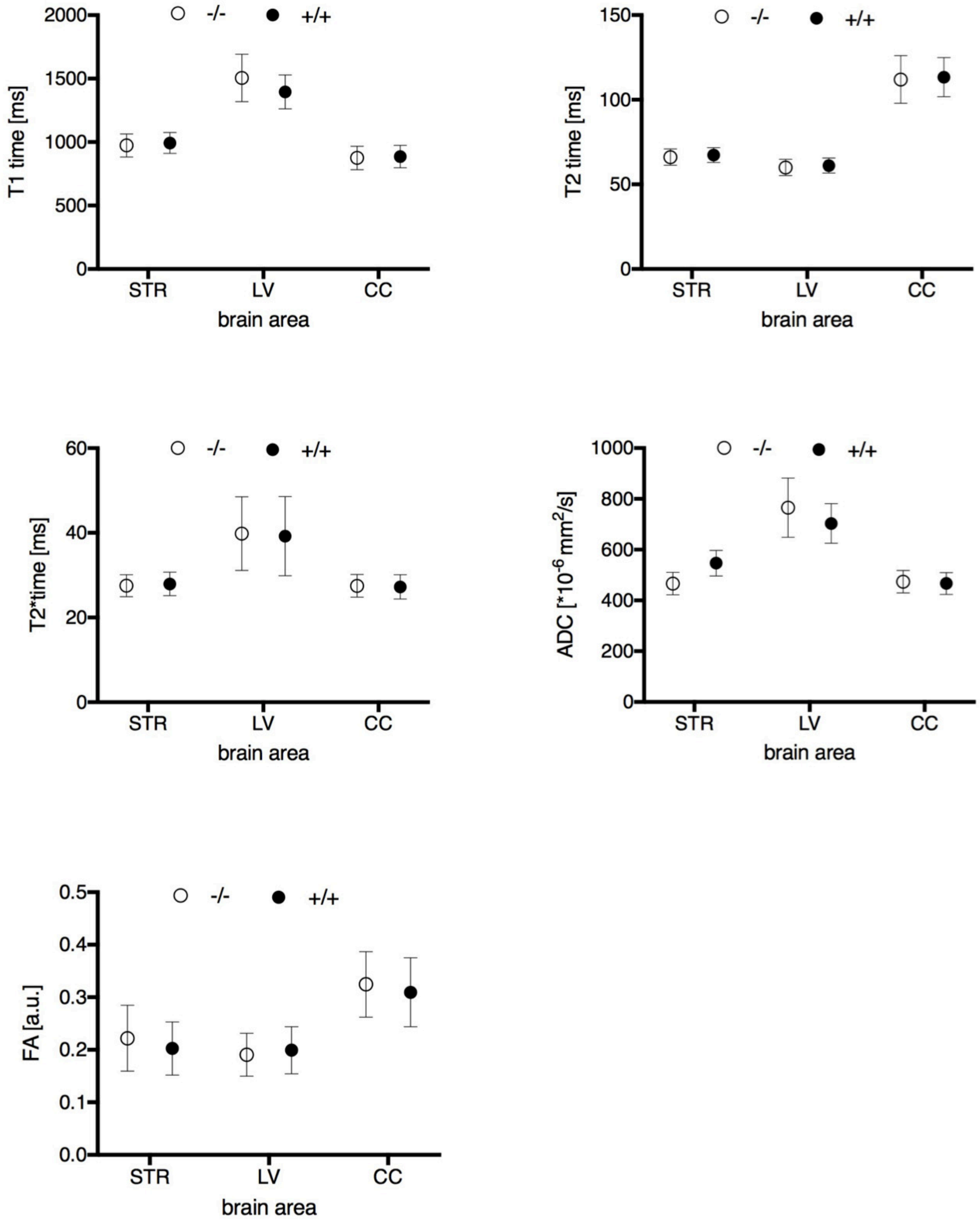

Supplementary Figure 4. T1-, T2- and T2*-relaxation times, apparent diffusion coefficient (ADC), and fractional anisotropy (FA) of striatum (STR), lateral ventricles (LV) and corpus callosum (CC) of wt (-/-) and tg (+/+) rats. No genotype-related significant differences were observed, yet striatal ADC values show a trend to be elevated in tg rats comparted to wt controls. All data are shown as mean ± SEM.
